# Global epidemiology of tick-borne *Alpharhabdovirinae*: a meta-analysis

**DOI:** 10.3389/fcimb.2026.1791903

**Published:** 2026-04-10

**Authors:** Yuqian Wu, Jingjing Zhang, Wenzhe Su, Zhoubin Zhang

**Affiliations:** 1 Guangzhou Center of Disease Control and Prevention (Guangzhou Health Supervision Institute), Guangzhou, China; 2 Institute of Public Health, Guangzhou Medical University, Guangzhou, China

**Keywords:** *Alpharhabdovirinae*, epidemiology, meta-analysis, systematic review, tick-borne disease, virus

## Abstract

**Introduction:**

The Alpharhabdovirinae subfamily of the family Rhabdoviridae encompasses a diverse and expanding group of tick-borne viruses, some of which pose potential risks as emerging human pathogens. Despite increasing detection through metagenomic surveillance, the global diversity, phylogenetic relationships, and taxonomic framework of tick-borne Alpharhabdovirinae (TBA) remain poorly characterized.

**Methods:**

This study conducted a comprehensive meta-analysis of all publicly available TBA sequences based on phylogenetic analysis of five structural proteins (N, P, M, G, L), combined with host associations and geographic distributions.

**Results:**

345 TBA strains were classified into 12 distinct phylogenetic clusters, each exhibiting unique evolutionary and ecological characteristics. These clusters include: (1) seven species-level lineages within the genus Alpharicinrhavirus, predominantly associated with Hyalomma and Haemaphysalis ticks across Eurasia; (2) a cluster related to Manly virus, widely distributed in Amblyomma, Haemaphysalis, and Rhipicephalus ticks acrossAustralia and China, exhibiting additional protein-coding genes of unknown function; (3) the genus Ledantevirus (21 species), characterized by broad host tropism including bats, rodents, and humans, with some members displaying phosphoprotein phylogenetic anomalies suggestive of recombination; (4) the genus Lostrhavirus, together with Tongliao Rhabd tick virus 1, forming a cluster associated with Hyalomma and Amblyomma ticks; (5) a Mononegavirus cluster comprising Alpharicinrhavirus heilongjiang, Alpharicinrhavirus skanevik (Norway mononegavirus 1), and Mononegavirales sp. specifically associated with Ixodesticks in Eurasia; and (6) one clusters with incomplete protein repertoires and uncertain taxonomic positions, including Tahe rhabdovirus 3 and Yanbian Rhabd tick virus 1 which lacks phosphoprotein entirely. This study provide a refined phylogenetic framework for TBA viruses, clarify their evolutionary relationships, and highlight critical knowledge gaps, including numerous uncharacterized hypothetical proteins and incomplete genomes that warrant further investigation.

**Discussion:**

This study underscores the importance of enhanced global surveillance and genomic characterization to assess the emergence potential and public health threat posed by this diverse group of tick-borne viruses.

## Introduction

The *Alpharhabdovirinae* subfamily is a well-established classification within the extensive *Rhabdoviridae* family, which comprises a diverse array of negative-sense (-) RNA viruses, including several significant pathogens (Walker et al., 2022a). The *Rhabdoviruses* were named for their bullet-like, cone-shaped, or bacilliform morphology (Kuzmin et al., 2009). Within the *Rhabdoviridae* family, the *Alpharhabdovirinae* subfamily stands as the largest, encompassing 31 genera of viruses that infect a wide range of hosts, including mammals, amphibians, reptiles, birds, fish, insects, ticks, and nematodes (Walker et al., 2022b). Three genera in this subfamily, *Alpharicinrhavirus*, *Ledantevirus*, and *Lostrhavirus* are known to be primarily associated with tick vectors. Tick-borne viruses outside these genera have been sporadically reported, such as Manly virus and Tahe rhabdoviruses, but these remain taxonomically unassigned at the genus level or represent putative novel lineages. This limited and fragmented taxonomic landscape underscores the need for comprehensive phylogenetic characterization to elucidate the true diversity and evolutionary relationships of tick-borne *Alpharhabdovirinae* (TBA).

The genomes of the *Alpharhabdovirinae* subfamily range in length within 10-16kb, encoding five structural proteins in a specific order: nucleocapsid protein (N), RNA-dependent RNA polymerase (L), matrix protein (M), glycoprotein (G), and phosphoprotein (P) (Kuzmin et al., 2009; Walker et al., 2022b). The nucleocapsid protein tightly encapsulates the entire viral RNA within an RNase-resistant core, serving as the template for both replication and transcription processes. The large, multi-functional RNA-directed RNA polymerase is responsible for both transcription and replication, which are crucial for viral assembly. The viral nucleocapsid is enveloped by a layer of matrix protein, forming virion particles and playing a role in mediating the inhibition of host gene expression. The transmembrane glycoprotein, located in the viral envelope, facilitates adsorption to host cell surface receptors.

Currently, ticks belong to the order *Ixodida*, classified into three families (*Ixodidae*, *Argasidae*, and *Nuttalliellidae*), including genera such as *Argas*, *Amblyomma*, *Dermacentor*, *Haemaphysalis*, *Hyalomma*, *Ixodes*, *Ornithodoros*, and *Rhipicephalus*. As blood-feeding vectors, ticks serve as ideal models for studying arbovirus transmission due to their distinct life stages and adaptability to diverse ecological environments. They undergo four life stages: egg, larvae, nymphs, and adults (Ni et al., 2023). Single-host ticks complete their entire life cycle on a single animal, while some ticks might feed on different animal hosts in different stages. The prevalence and geographical distribution of ticks are expanding, and they maintain and transmit a wide spectrum of viral pathogens between humans and animals. Consequently, ticks are vectors of emerging and re-emerging viruses, making tick-borne viruses a significant global health concern (Paules et al., 2018).

High-throughput sequencing has revealed the presence of *Alpharicinrhaviruses* within the *Alpharhabdovirinae* subfamily in hard ticks belonging to the *Ixodidae* family, possessing all five canonical rhabdovirus structural protein genes (N, P, M, G, and L) (Walker et al., 2022b). Notably, *Alpharicinrhavirus bole* (Bole Tick Virus 2) was initially identified from *Hyalomma asiaticum* ticks in Bole, Xinjiang Province, China, in 2012, and *Alpharicinrhavirus wuhan* (Wuhan Tick Virus 1, WHTV1) was identified from *Rhipicephalus microplus* ticks in Wuhan, Hubei Province, China (Li et al., 2015). In 2019, another *Alpharicinrhavirus* was detected in Hubei Province and designated as *Alpharicinrhavirus hubei* (Hubei Tick Rhabdovirus 1, HTRV) (Xu et al., 2021). Additionally, *Alpharicinrhavirus blanchseco* was identified in *Amblyomma ovale* ticks in the Caribbean twin-island Republic of Trinidad and Tobago in 2017 (Sameroff et al., 2019). To date, the genus comprises 17 officially recognized species identified from ticks (Pettersson et al., 2017; Gofton et al., 2022; Sameroff et al., 2022).

*Ledanteviruses* comprise a diverse group of rhabdoviruses that infect a broad range of mammalian hosts, including bats, rodents, cattle, and humans. Ticks are considered the primary vectors for most members of this genus, although the transmission routes for some species remain to be fully elucidated. A distinctive genomic feature of certain *Ledanteviruses* is the presence of an additional gene of unknown function (Walker et al., 2022b). The genus was first established following the isolation of Le Dantec virus (now known as *L. ledantec*) from a patient in Senegal in 1965 (Blasdell et al., 2015). Subsequently, a growing number of related viruses were identified from ticks and mammals across Africa and Asia, including Fikirini rhabdovirus (*L. fikirini*) (Kading et al., 2013) and Kolente virus (*L. kolente*) (Ghedin et al., 2013). The discovery of these viruses, together with several novel bat-associated rhabdoviruses, led to the formal proposal of *Ledantevirus* as a new genus within the family *Rhabdoviridae* (Blasdell et al., 2015). This taxonomic framework was further supported by the characterization of Kumasi rhabdovirus (*L. kumasi*) from bats in Ghana (Binger et al., 2015). To date, the genus comprises 21 officially recognized species identified from diverse vertebrate and arthropod hosts across multiple continents (Walker et al., 2015; Li et al., 2015; Lelli et al., 2018; Bennett et al., 2020).

*Lostrhaviruses* have been detected in hard ticks and may possess an open reading frame (ORF) with an unknown function (Walker et al., 2022b). The *Lostrhavirus* genus was initially isolated from *Amblyomma americanum*, commonly known as the lone star tick, and was subsequently named after this tick. Currently, there are three recognized species within this genus: *Lostrhavirus lonestar* found in the United States, *Lostrhavirus hyalomma* discovered in China, and *Lostrhavirus alxa* from China (Kong et al., 2022).

Additional novel viruses belonging to the *Alpharhabdovirinae* subfamily have been identified in association with ticks, yet they lack comprehensive genus and species annotations. Notably, the manly virus was from *Amblyomma moreliae* in Australia in 2016 and from *Haemaphysalis concinna* in China in 2020 (Harvey et al., 2019; Guo et al., 2022). A closely related species, the Guangdong tick manly virus, has also been reported (Qin et al., 2023).

Summarizing the information on tick-associated viruses within the *Alpharhabdovirinae* subfamily, along with recently discovered tick-borne viruses that exhibit close relatedness to *Alpharhabdovirinae* but remain insufficiently annotated, is of significant importance. These findings have the potential to enhance our understanding of tick-borne *Alpharhabdovirinae* (TBA) and can contribute to a better comprehension of virus transmission among ticks and other hosts.

## Materials and methods

### Searching strategy and selection criteria

The nucleotide and amino acid sequences of tick-borne viruses belonging to the *Alpharhabdovirinae* subfamily were downloaded from the NCBI database in April, 2025. This initial dataset specifically included sequences from the genera *Alpharicinrhavirus*, *Ledantevirus*, and *Lostrhavirus*. Additionally, sequences of various other tick-borne viruses were extracted from NCBI (**Table 1**), retrieved using the results of a BlastP search with the sequences obtained in the previous step serving as the query.

**Table 1:** Taxonomy of species found by BlastP.

Species	Taxonomy ID	Kingdom	Phylum	Class	Order	Family	Genus	Reference
Guangdong tick manly virus	2998221	–	–	–	–	–	–	Guo et al., 2022
Manly virus	2485873	–	–	–	–	–	–	Harvey et al., 2019
Mononegavirales sp.	1955139	Orthornavirae	Negarnaviricota	Monjiviricetes	Mononegavirales	–	–	Cross et al., 2018
Nayun tick rhabdovirus	1610818	Orthornavirae	Negarnaviricota	Monjiviricetes	Mononegavirales	Rhabdoviridae	–	Xia et al., 2015
Qingyang Rhabd tick virus 1	2972324	Orthornavirae	Negarnaviricota	Monjiviricetes	Mononegavirales	Rhabdoviridae	–	Ni et al., 2023
Rhipicephalus associated rhabdo-like virus	2569609	Orthornavirae	Negarnaviricota	Monjiviricetes	Mononegavirales	Rhabdoviridae	–	Shi et al., 2021
Tacheng tick virus 3	1608085	Orthornavirae	Negarnaviricota	Monjiviricetes	Mononegavirales	Rhabdoviridae	–	Li et al., 2015
Tahe rhabdovirus 3	2983976	Orthornavirae	Negarnaviricota	Monjiviricetes	Mononegavirales	Rhabdoviridae	–	Liu et al., 2022
Tongliao Rhabd tick virus 1	2972331	Orthornavirae	Negarnaviricota	Monjiviricetes	Mononegavirales	Rhabdoviridae	–	Ni et al., 2023
Tongren Rhabd tick virus 3	2972337	Orthornavirae	Negarnaviricota	Monjiviricetes	Mononegavirales	Rhabdoviridae	–	Ni et al., 2023
Yanbian Rhabd tick virus 4	2972328	Orthornavirae	Negarnaviricota	Monjiviricetes	Mononegavirales	Rhabdoviridae	–	Ni et al., 2023
Yanbian Rhabd tick virus 5	2972339	Orthornavirae	Negarnaviricota	Monjiviricetes	Mononegavirales	Rhabdoviridae	–	Ni et al., 2023

For entries marked with “-”, the taxonomic information (Phylum, Class, Order, Family, Genus) is currently unavailable in the NCBI Taxonomy database or the ICTV Virus Taxonomy database due to insufficient characterization or pending official classification.

**Figure 1:**
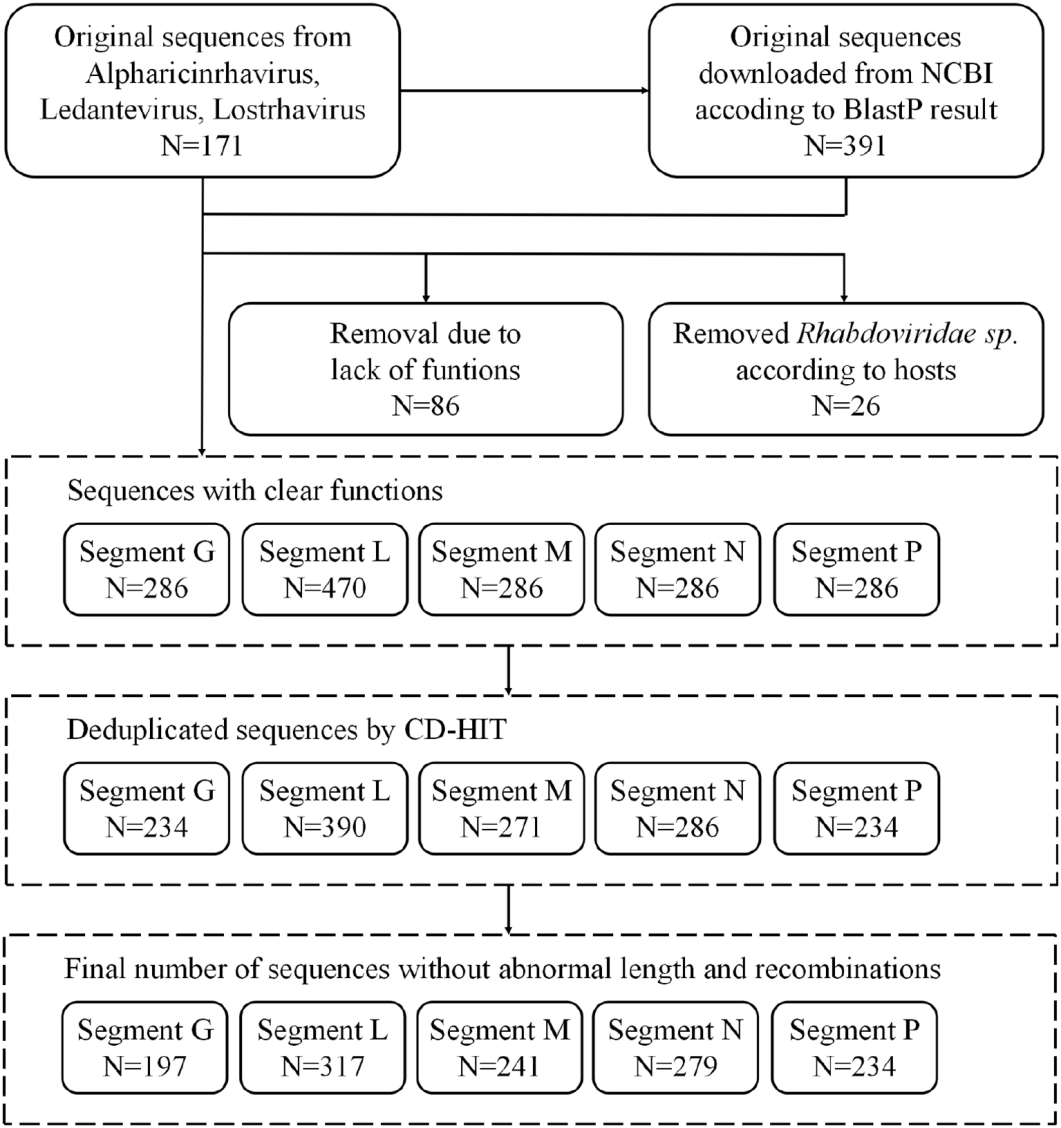
Flowchart of data selection process in this study.

CDS (coding DNA sequences) and their corresponding amino acid sequences were extracted. These sequences were subsequently classified into five types - nucleoprotein (N), phosphoprotein (P), matrix protein (M), glycoprotein (G), and RNA-dependent RNA polymerase (L) - based on their annotations in NCBI (**Figure 1**). Notably, some records were labeled as “hypothetical protein” rather than bearing the aforementioned specific annotations. For the well-annotated proteins, they served as subjects in a BlastP analysis, where the hypothetical proteins were queried to determine their most probable annotations.

Given that the focus of this analysis is on tick-borne viruses within the *Alpharhabdovirinae* subfamily of the *Rhabdoviridae* family, sequences unrelated to ticks were excluded based on their host annotations. Keywords used to identify tick hosts included various tick species and genera such as “Am.”, “Amblyomma”, “Anocentor”, “Aponomma”, “Argas”, “Boophilus”, “Dermacetor” (typo for “Dermacentor”), “Dermacentor”, “Haemaphysalis”, “Haemophysalis” (typo for “Haemaphysalis”), “Histiostoma”, “Hyalomma”, “Ixodes”, “Ixodides”, “Ixodus” (typo for “Ixodes”), “Margaropus”, “Nuttalliella”, “Ornithodoros”, “Otobius”, “Rh.”, “Rhipicentor”, “Rhipicephalus”, “Ripicephalus” (typo for “Rhipicephalus”), “Smithiella”, and “tick” (the general term). The remaining sequences were utilized for further analysis.

### Data analysis

CD-HIT was utilized to de-duplicate CDS sequences with 100% identity (Li and Godzik, 2006). To ensure the inclusion of sequences with an appropriate length in this study, full-length complete sequences served as the reference. For each species within each protein type, the mean (μ) and standard deviation (σ) of the sequence lengths were calculated. Only sequences exceeding 0.8 * μ and not surpassing μ + 3σ were considered for further analysis. MAFFT was employed to align the amino acid sequences (Katoh and Standley, 2013). Subsequently, PAL2NAL was used to align the CDS sequences based on the amino acid sequence alignment (Suyama et al., 2006). Sequences potentially exhibiting recombination effects, detected by RDP4, were excluded from subsequent analyses (Martin et al., 2015).

TrimAL was utilized to remove regions with gaps in more than 30% of the sequences in the alignment (Capella-Gutierrez et al., 2009). MAFFT was then used to combine and align amino acid sequences from various species within the same protein type. Following this, PAL2NAL aligned the CDS sequences based on the amino acid sequences, with gap regions removed according to the previous step. Both amino acid and CDS sequences were purged of regions that were unique to a single species but were gapped in any other species.

IQ-Tree2 (version 2.2.6, with GTR+I+G model) was employed to construct phylogenetic trees for each protein type (Minh et al., 2020). The trees were re-rooted using the midpoint outgroup method provided by the ete3 Python package (Huerta-Cepas et al., 2016). The visualization of the phylogenetic trees was facilitated by the R packages treeio, ggtreeExtra, and ggtree (Wang et al., 2020; Xu et al., 2021; Yu, 2020).

Downstream analysis classified viruses into clusters based on their phylogeny across all five protein types. Phylogenetic clusters were defined based on the principle of monophyly. Specifically, a cluster was recognized when a group of sequences consistently formed a monophyletic lineage with high bootstrap support in at least three of the five protein trees (N, P, M, G, L). Clusters were further supported by distinct host associations and/or geographic distributions. Notably, the term “cluster” is used here as an operational taxonomic unit that may correspond to various taxonomic ranks: (i) established viral genera when all members form a monophyletic group; (ii) established viral species when they constitute distinct monophyletic lineages; and (iii) putative novel species or higher-level lineages for previously unclassified viruses that form monophyletic groups distinct from known taxa. This phylogeny-driven approach enables comprehensive characterization of TBA diversity beyond existing taxonomic constraints.

## Result

Based on the phylogenetic analysis of all five structural proteins (N, P, M, G, L), distinct and well-supported lineages were identified. Each species forms a monophyletic cluster with high bootstrap support across multiple protein trees, and all strains within these clusters are exclusively associated with tick hosts, reinforcing the close evolutionary relationship hosts. The following sections describe the phylogenetic characteristics, host ranges, and geographic distributions of each species in detail.

### Alpharicinrhavirus

All strains of *Alpharicinrhavirus* have been identified from ticks, underscoring the close relationship between this virus genus and its hosts. Of the recorded *Alpharicinrhavirus* isolates, the majority were collected in China, with notable exceptions including *A. blanchseco* TTP-Pool-17 (NC_076415/MN025503) from Trinidad and Tobago, *A. bole* MU5 (MG764527) from Turkey, *A. wuhan* Thailand tick rhabdovirus (MN095536) from Thailand, and *A. wuhan* MU28 (MG764528) also from Turkey. Notably, *A. blanchseco* is the only *Alpharicinrhavirus* species known to infect *Amblyomma ovale* ticks, and only one isolate was analyzed in this study due to the identity of sequences NC_076415 and MN025503. *A. bole*, also known as Bole tick virus 2, has been detected in Hyalomma ticks since 2015, predominantly in *H. asiaticum* but occasionally in *H. scupense* and *H. marginatum*.

**Figure 2:**
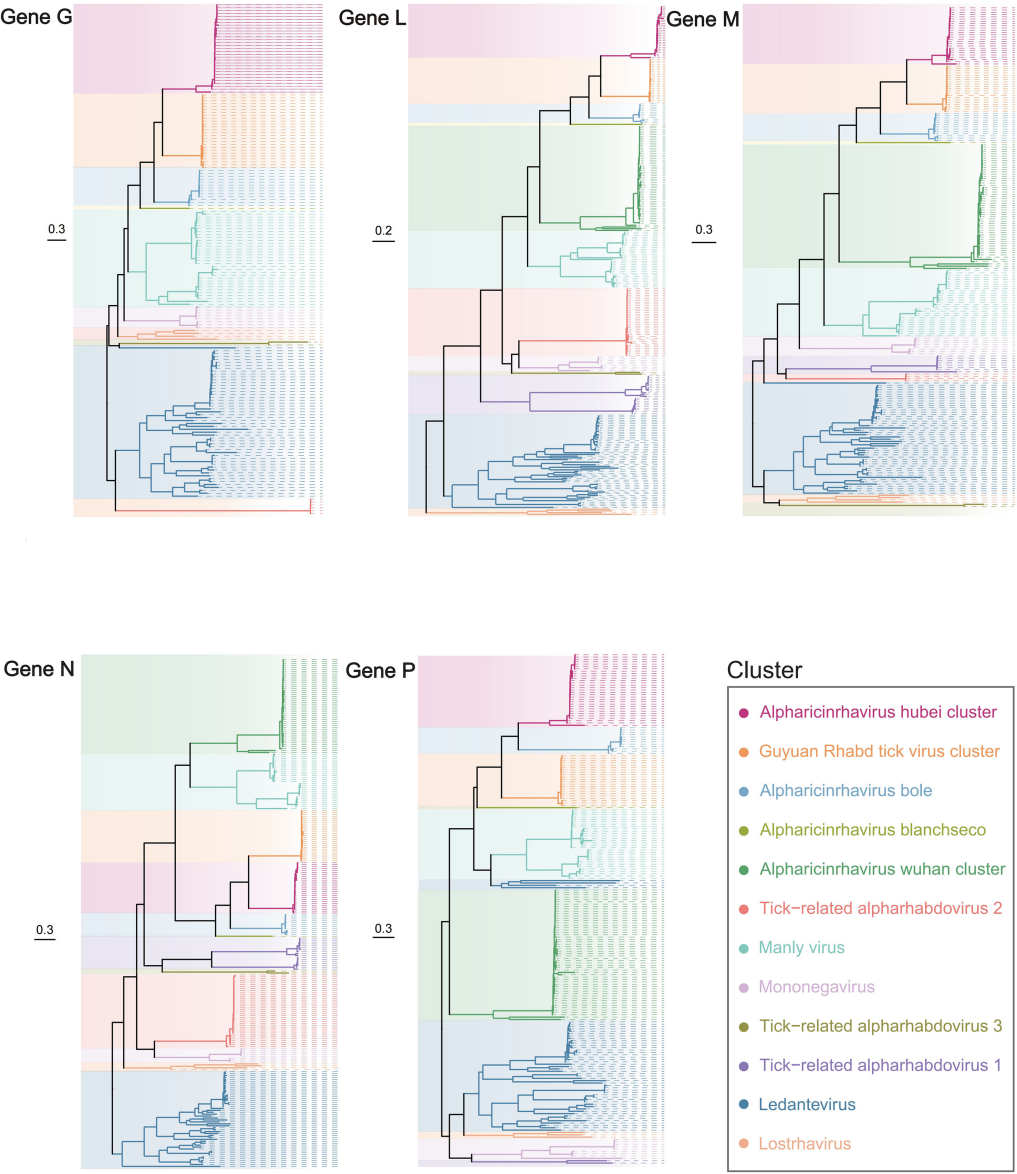
Clusters of TBAs. The phylogenetic tree was constructed based on the concatenated amino acid sequences of five structural proteins (glycoprotein [G], RNA-dependent RNA polymerase [L], matrix protein [M], nucleoprotein [N], and phosphoprotein [P]) using the maximum likelihood method implemented in IQ-Tree2. The tree was rooted using the midpoint rooting method. Scale bars wshown at bottom).Colored strips and shaded backgrounds denote the 12 distinct phylogenetic clusters identified in this study, each defined by consistent monophyly across multiple protein trees, host associations, and geographic distributions. Detailed composition of each cluster is provided in **Table 2**.

A single record of *A. hubei* was retrieved from NCBI. Phylogenetic analysis revealed that Qingyang Rhabd tick virus 1, Tongren Rhabd tick virus 3, and certain Rhabdoviridae species share a close phylogenetic relationship with *A. hubei* (**Figure 2**). All viruses within this cluster were identified from *Haemaphysalis* ticks after 2018, primarily *H. longicornis* but also occasionally in *H. japonica* and *H. hystricis*. Unlike other strains in this cluster, Qingyang Rhabd tick virus 1 contains an additional gene encoding a hypothetical protein.

*Alpharicinrhavirus ningxia*, previously known as Guyuan Rhabd tick virus 1, along with three Rhabdoviridae species, formed a cluster. All 27 strains within this species were discovered in Guyuan, Ningxia Province, China, from *Haemaphysalis* ticks, specifically *H. japonica*, *H. longicornis*, and *H. qinghaiensis*. In phylogenetic trees of *Alpharicinrhavirus*, this cluster exhibited monophyly. Notably, strains such as ON746521.1 encode six proteins, rather than the typical five encoded by most TBA; however, the function of the additional protein remains unknown.

*Alpharicinrhavirus bancrofti* from *Haemaphysalis bancrofti* ticks in Australia and *Alpharicinrhavirus huangpi* from *Haemaphysalis doenitzi* ticks in China formed the same cluster. In the phylogenetic tree based on matrix proteins, this cluster is closely related to *Lostrhavirus*.

### Manly virus

Manly virus has been identified from ticks in both Australia and China (Harvey et al., 2019; Qin et al., 2023). *Alpharicinrhavirus huanggang*, *Alpharicinrhavirus nanning*, *Alpharicinrhavirus tahe*, Guangdong tick Manly virus, Manly virus, Yanbian Rhabd tick virus 4, Yanbian Rhabd tick virus 5, and several *Rhabdoviridae* sp. clustered in phylogenetic trees. All strains within this cluster were identified from ticks, specifically *Amblyomma moreliae*, *Amblyomma varanense*, *Haemaphysalis concinna*, *Haemaphysalis japonica*, and *Rhipicephalus microplus*. With the exception of Manly virus MK026564, which was identified in Australia in 2016, all other strains were identified since 2017 in southeast China (Guangdong, Guangxi, Fujian, and Hubei) and northeast China (Heilongjiang, Hebei, and Jilin). Notably, strains such as *A. huanggang* (ON746518.1) encode six proteins; however, the function of the additional protein remains unknown.

### Ledantevirus

According to the NCBI taxonomy database, the genus *Ledantevirus* comprises 20 species, including *L. barur*, *L. bughendera*, *L. elgon*, *L. fikirini*, *L. fukuoka*, *L. kanyawara*, *L. kern*, *L. keuraliba*, *L. kolente*, *L. kumasi*, *L. ledantec*, *L. longquan*, *L. nishimuro*, *L. nkolbisson*, *L. oita*, *L. taiyi*, *L. vaprio*, *L. wenzhou*, *L. wuhan*, and *L. yongjia*. Additionally, Bat ledantevirus, Jingmen bat ledantevirus 1, Odro virus, and Wufeng rodent ledantevirus 1 also belong to this genus. Based on the phylogenetic trees of the G, L, M, and N proteins, three *Rhabdoviridae* sp. strains (NC_076186, ON812533, OR868987) may be related to *Ledantevirus*. However, in the phylogenetic tree of the phosphoprotein, these three strains and *L. kumasi* (Kumasi rhabdovirus) were closer to Manly virus and *Alpharicinrhavirus* than to *Ledantevirus* (**Figure 2**). Although most genes in *Ledanteviruses* have been well annotated, some remain unknown; for instance, three genes in *L. kumasi* encode hypothetical proteins. Among these, YP_009177010.1 and AIL31434.1 are likely part of the phosphoprotein, but their phylogenetic relationships may not be accurately represented. Other *Ledanteviruses*, such as *L. fukuoka*, *L. kern*, *L. keuraliba*, *L. ledantec*, *L. vaprio*, and Odro virus, encode one extract protein with an unknown function. Conversely, some *Ledanteviruses*, like Jingmen bat ledantevirus, have incomplete genomes and only encode the L protein.

*Ledantevirus* can be identified from ticks such as *Haemaphysalis hystricis* and various species of *Rhinolophus* bats such as *R. affinis*, *R. cornutus*, *R. eloquens*, *R. pusillus*, and *R. sinicus* in China, Japan, and Kenya. Additionally, *Ledantevirus* can be identified from other mammals (including rats and wild pigs) and arthropods (such as fleas, flies, and midges) in Cameroon, China, Ghana, India, Japan, Kenya, Uganda, the USA, and Senegal. Cases of *Ledantevirus* isolation from humans, such as *L. ledantec*, have also been reported, demonstrating the virus’s multiple host tropism and potential risk (Ashraf et al., 2023).

### Lostrhavirus

According to the NCBI taxonomy database, the genus *Lostrhavirus* comprises only three species: *L. alxa* and *L. hyalomma* from *Hyalomma asiaticum* in China, and *L. lonestar* from *Amblyomma americanum* in the USA. Based on phylogenetic analysis, Tongliao Rhabd tick virus 1 identified from *Dermacentor silvarum* in Inner Mongolia, China, exhibits close relatedness to *Lostrhavirus* and possesses a gene encoding a hypothetical protein, UYL95600.1, which is postulated to be its phosphoprotein.

### Mononegavirus

*Alpharicinrhavirus skanevik* (Norway mononegavirus 1), *Alpharicinrhavirus heilongjiang* (Tahe rhabdovirus 2), and a *Mononegavirales* sp. strain from *Ixodes persulcatus* in China and *Ixodes ricinus* in Norway collectively formed a viral cluster. In phylogenetic trees based on protein G and M sequences, the strains within this cluster exhibited close relatedness to those within the *Alpharicinrhavirus* genus. However, they did not display close relatedness in the phylogenetic tree based on phosphoprotein sequences. Within this cluster, OP863286 from *I. persulcatus* in China was initially classified as belonging to other *A. skanevik* strains from *I. ricinus* in Norway (Qin et al., 2023).

### Other tick-related alpharhabdovirus

*Alpharicinrhavirus taishun*, *Alpharicinrhavirus zhangjiakou*, and several *Rhabdoviridae* sp. strains clustered. Within this cluster, the L and N proteins of the viruses can be identified. However, matrix proteins remain unidentifiable in viruses such as *A. taishun*. Additionally, phosphoprotein and glycoprotein have not been detected in any of the strains within this cluster. The viruses within this cluster can be further differentiated into two sub-clusters. The first sub-cluster contains solely *A. taishun* isolates, derived from ticks such as *Hyalomma asiaticum*, *Hyalomma detritum*, *Haemaphysalis hystricis*, and *Hyalomma scupense*. The second sub-cluster comprises strains from *A. zhangjiakou* and all *Rhabdoviridae* sp. within this cluster, which were collected from *D. silvarum* in China between 2018 and 2019 (nine out of ten isolates were from Zhangjiakou, Inner Mongolia province). *A. taishun* produces hypothetical proteins that may belong to the M and N protein families, although these have not yet been thoroughly annotated.

Tahe rhabdovirus 3, Yanbian Rhabd tick virus 1, and multiple *Rhabdoviridae* sp. strains, all from *I. persulcatus* ticks in northeastern Chinese provinces such as Inner Mongolia, Heilongjiang, and Jilin, formed another cluster. In comparison to other strains within the *Alpharhabdovirinae* subfamily, the strains in this cluster are capable of producing only four proteins and lack the phosphoprotein. Certain species, including Tahe rhabdovirus 3 and Yanbian Rhabd tick virus 1, possess hypothetical proteins that may correspond to the M and N protein families. In phylogenetic trees based on L and N proteins, this cluster exhibits close relatedness to the Mononegavirus cluster, and both share the Ixodes tick as a host.

## Discussion

### Phylogenetic suggestions

According to the number of proteins that were well annotated or identified, the phylogenetic trees of each protein type, the host taxonomy, and the collection site, TBA can be classified into 12 clusters (**Table 2**). It should be noted that the phylogenetic clusters identified in this study represent monophyletic lineages based on molecular data and operational criteria, which may not strictly align with formal taxonomic ranks defined by the ICTV. Nevertheless, this phylogeny-driven framework provides a useful reference for understanding the evolutionary relationships and diversity of TBA viruses, and may inform future taxonomic revisions.

**Table 2:** Clusters and species of TBA.

Cluster name	Species included in each cluster	Number of sequences finally used in each cluster				
		G protein	L protein	M protein	N protein	P protein
Blanchseco	*A. blanchseco*	1	1	1	1	1
Bole	*A. bole*	13	12	13	11	12
Guyuan	*A. ningxia*, *Rhabdoviridae* sp. (ON812476, ON812572, ON812573)	26	26	21	27	22
Huangpi	*A. bancrofti*, *A. huangpi*	2	2	2	2	2
Hubei	*A. hubei*, Qingyang Rhabd tick virus 1, Tongren Rhabd tick virus 3, *Rhabdoviridae* sp. (MW721935, MW721937, MW721939, MW721940, MZ965022, MZ965023, MZ965024, NC_076504, ON812477, ON812529, ON812530, ON812531, ON812532, ON812545, ON812546, ON812547, ON812548, ON812555, ON812556, ON812559, ON812560, ON812561, ON812562, ON812568, ON812569, ON812570, ON812575, PP945082, PP945083, PP945084)	31	31	26	27	31
Ledante	Bat ledantevirus, Jingmen bat ledantevirus 1, *L. barur*, *L. bughendera*, *L. elgon*, *L. fikirini*, *L. fukuoka*, *L. kanyawara*, *L. kern*, *L. keuraliba*, *L. kolente*, *L. kumasi*, *L. ledantec*, *L. longquan*, *L. nishimuro*, *L. nkolbisson*, *L. oita*, *L. taiyi*, *L. vaprio*, *L. wenzhou*, *L. wuhan*, *L. yongjia*, Odro virus, *Rhabdoviridae* sp. (NC_076186, ON812533, OR868987), Wufeng rodent ledantevirus 1	52	55	50	50	51
Lostrha	*L. alxa*, *L. hyalomma*, *L. lonestar*, Tongliao Rhabd tick virus 1	4	4	4	4	3
Manly	*A. huanggang*, *A. nanning*, *A. tahe*, Guangdong tick manly virus, Manly virus, *Rhabdoviridae* sp. (ON812474, ON812480, ON812481, ON812512, ON812513, ON812514, ON812515, ON812516, ON812517, ON812518, ON812522, ON812524, ON812525, ON812526, ON812528, ON812549, ON812550, ON812552, ON812553, ON812554, ON812557, ON812574), Yanbian Rhabd tick virus 4, Yanbian Rhabd tick virus 5	33	33	30	29	30
Mononega	*A. heilongjiang*, *A. skanevik*, *Mononegavirales* sp.	8	10	8	9	9
Wuhan	*A. wuhan*, *A. reticulatus*, *A. qinghai*, Nayun tick rhabdovirus, Rhipicephalus associated rhabdo-like virus, Tacheng Tick Virus 3	0	62	56	49	56
Yanbian	*Rhabdoviridae* sp. (ON812475, ON812482, ON812519, ON812520, ON812521, ON812563, ON812566, ON812567, OR148806, OR148807, OR148808, OR148809, OR148810, OR148811, OR148812, OR148815, OR148816, OR148817, OR148818, OR148819, OR148820, OR148821, OR148822, OR148823, OR148824, OR148825, OR148826, OR148827, OR148828, OR148829, OR148830, OR148831, OR148832), Tahe rhabdovirus 3, Yanbian Rhabd tick virus 1	6	39	4	37	0
Zhangjiakou	*A. taishun*, *A. zhangjiakou*, *Rhabdoviridae* sp. (ON812484, ON812539, ON812540, ON812541, ON812542, ON812543, ON812571, ON812576, ON872648)	0	23	8	17	0
Total number		176	298	223	263	217

Phylogenetic analysis revealed that *A. hubei*, Qingyang Rhabd tick virus 1, Tongren Rhabd tick virus 3, and certain *Rhabdoviridae* species can be grouped into a cluster termed the “Hubei cluster”. *A. ningxia*, along with three Rhabdoviridae species, formed a distinct cluster termed the “Ningxia cluster”. *A. huanggang*, *A. nanning*, *A. tahe*, Guangdong tick Manly virus, Manly virus, Yanbian Rhabd tick virus 4, Yanbian Rhabd tick virus 5, and several *Rhabdoviridae* sp. formed a viral cluster, termed the “Manly cluster”. This cluster exhibits monophyly in the phylogenetic trees of *Alpharicinrhavirus*, particularly in the phylogenetic tree of the phosphoprotein, indicating that the strains from the Manly virus cluster can be considered as a clade within *Alpharicinrhavirus*. Based on phylogenetic analysis, *Lostrhavirus* and Tongliao Rhabd tick virus 1 can be grouped into a viral cluster termed the “Lostrha cluster”. *A. skanevik*, *A. heilongjiang*, and a *Mononegavirales* sp. strain from *I. persulcatus* in China and *I. ricinus* in Norway collectively formed a viral cluster designated as the “Mononega cluster”. Several viruses could potentially be classified within the *Alpharhabdovirinae* subfamily.

Except the viruses mentioned above, based on the number of identifiable protein functions and the phylogeny of each protein, other tick-related *Alpharhabdovirus* can be categorized into three clusters, as outlined in **Table 2**. The Zhangjiakou cluster comprises *A. taishun*, *A. zhangjiakou*, and several *Rhabdoviridae* sp. strains. Yanbian cluster comprises Tahe rhabdovirus 3, Yanbian Rhabd tick virus 1, and multiple *Rhabdoviridae* sp. strains. Huangpi cluster includes *A. bancrofti* and *A. huangpi*, which is close to Zhangjiakou cluster in the nucleoprotein tree, and is close to the Mononega cluster in the phosphoprotein tree. The phylogeny of the strains within Huangpi cluster is complex, suggesting the possibility of recombination among strains within this cluster and even within the *Alpharicinrhavirus* genus.

Certain TBA exhibit a close relationship with specific, limited tick species. For instance, viruses associated with *A. bole* have only been identified in three species of *Hyalomma* ticks, while viruses related to *A. hubei* have been identified from five species of *Haemaphysalis* ticks. The hosts of viruses within the Guyuan cluster and Huangpi cluster are confined to three species of *Haemaphysalis*. Mononega cluster and Yanbian cluster are specifically associated with *I. ricinus* and *I. persulcatus* ticks. The strong correlation between virus clusters and tick species may be underpinned by co-selection between viruses and their hosts, which warrants further investigation.

Three *Rhabdoviridae* sp. strains (NC_076186, ON812533, OR868987) and *L. kumasi* (Kumasi rhabdovirus) were closer to *Alpharicinrhavirus* than to *Ledantevirus*, suggesting the possibility of recombination or anomalies in the phosphoprotein of *L. kumasi*.

The viruses within Manly cluster also form a monophyletic group that is closely related to the *Alpharhabdovirus* genus, and all of these viruses were from ticks. This underscores the importance of incorporating viruses from the manly virus cluster into studies of *Alpharhabdovirus*, particularly in investigations related to phylogeny and ecology, as it can aid in enhancing our understanding of the host adaptability of viruses within ticks.

This study has revealed that certain strains, despite being classified within the same species, may be more accurately designated as separate species. For instance, within *A. skanevik*, strain HLJ-IP-6 (OP863286.1) exhibits distinct isolation sites and hosts compared to strains NOR_A2_Bronnoya_2014 (MF141072.1) and NOR_H3_Skanevik_2014 (MF141073.1). Based on phylogenetic analysis, these strains are separated, suggesting they may belong to different species. Given the differences in their isolation countries, hosts, and phylogenetic patterns, particularly in protein L and N, OP863286 may warrant classification as a novel species. Similarly, in Manly virus, strain MK026564.1 displays different isolation sites and hosts compared to strain OP863293.1, and phylogenetic separation indicates they too may constitute distinct species.

### Experimental requirements and researches for further analysis

Further in-depth analysis of specific genes and their associated products within the TBA is necessary. While the majority of TBA possess five genes encoding the G, L, M, N, and P proteins, certain members, such as Tahe rhabdovirus 3 and Yanbian Rhabd tick virus 1 within Yanbian cluster, still exhibit products with undefined functionalities. In Zhangjiakou cluster, sequences annotated as “hypothetical protein” have been identified, which may represent potential matrix protein, phosphoprotein, and glycoprotein candidates; however, further experimental validation is required. Although this study has identified some hypothetical products with potential roles, experimental validation is still required for confirmation. Additionally, some TBA contain extra proteins encoding hypothetical proteins of unknown significance, necessitating further investigation and research. Studying these proteins could enhance our understanding of TBA transmission, host fitness, and virulence; however, there is a scarcity of related research in this area. Notably, many viruses within TBA, including all *Alpharicinrhaviruses*, have yet to be identified, which complicates the analysis of their proteins. Furthermore, some TBA, like Jingmen bat ledantevirus, lack a reported complete genome, impeding the study of phylogeny based on their entire genome and necessitating further strain collection.

At least four strains related to *L. kumasi* exhibit unusual phylogeny in their phosphoproteins. This anomaly may be attributed to potential recombination events involving the phosphoprotein or to instability within the phosphoprotein of these strains. Further research is required to determine the precise underlying cause.

In addition to ticks serving as hosts, some TBA within Ledante cluster and Mononega clusters also infect animals and, potentially, humans (Walker et al., 2015). Notably, several novel TBA have been reported in limited studies conducted in China (Li et al., 2015; Ni et al., 2023), highlighting the significance of conducting high-throughput screening for tick-borne viruses globally. It is anticipated that similar studies worldwide will uncover more TBA, thereby expanding our knowledge about tick-borne viruses and our understanding of the threats posed by potential pathogens. Such studies may identify infectious TBA harmful to humans and livestock, ultimately contributing to public health protection.

Future studies on TBA viruses can adopt multi-layered experimental approaches similar to those employed in recent toxicological research. Recent studies combined systematic data mining with focused experimental validation (Chen et al., 2026), which could be adapted to investigate the functional significance of specific TBA viral proteins, particularly those with phylogenetic anomalies or unknown functions. The molecular docking and dynamics simulations for interaction validation (Shang et al., 2025) demonstrate the value of computational approaches in studying virus-host protein interactions. Such interdisciplinary methodologies will be essential for elucidating the pathogenic potential of TBA viruses and their implications for public health.

### Importance of tick-borne disease analysis

Our findings underscore the ongoing public health threat posed by emerging tick-borne viruses in subtropical urban environments. This threat is exemplified by the recent discovery of novel viruses like the Wetland virus (WELV) in China, which is associated with human febrile illness and has been detected in multiple tick species and domestic animals (Yuan et al., 2025). In addition, systematic surveillance in regions such as Inner Mongolia has identified a diverse array of tick-borne viruses, including several newly described ones like Meitian tick virus (MtTV), Ilekde tick virus (YLTV), and Songling virus (SGLV), highlighting a much richer and largely uncharacterized viral landscape (Zhang et al., 2025).

The spread of these viruses is facilitated by multiple factors. Migratory birds act as long-distance transporters for both ticks and the pathogens they carry, a dynamic evident in marine ecosystems and relevant to the introduction of viruses into new geographic areas (Capasso et al., 2024). Concurrently, climate change and rapid urbanization are expanding suitable habitats for vector mosquitoes like *Aedes albopictus*. These factors collectively increase opportunities for enzootic maintenance in peri−urban areas and subsequent spillover into human populations. Enhanced surveillance targeting ticks, animal reservoirs, and human febrile illnesses is therefore critically warranted to assess the full spectrum of circulating arboviruses and their epidemic potential.

### Summary

This study clustered TBA based on the phylogenies of their genes in current research and identified several characteristics among these strains, underscoring the importance of studies on tick-borne diseases. Further research is necessary to elucidate the functions of TBA products and to monitor novel TBA worldwide, thereby better defining the risks they may pose to human and animal health.

## Data Availability

Publicly available datasets were analyzed in this study. The data can be found in NCBI Nucleotide database.
