# Supplementary material for: Global epidemiology of tick-borne Alpharhabdovirinae: a meta-analysis
Source: Front Cell Infect Microbiol. 2026 Apr 10;16:1791903. doi: 10.3389/fcimb.2026.1791903 (PMC13106086; doi:10.3389/fcimb.2026.1791903)
Supplement: Supplementary file 1 [file DataSheet1.pdf]

S1 Fig. Gene G

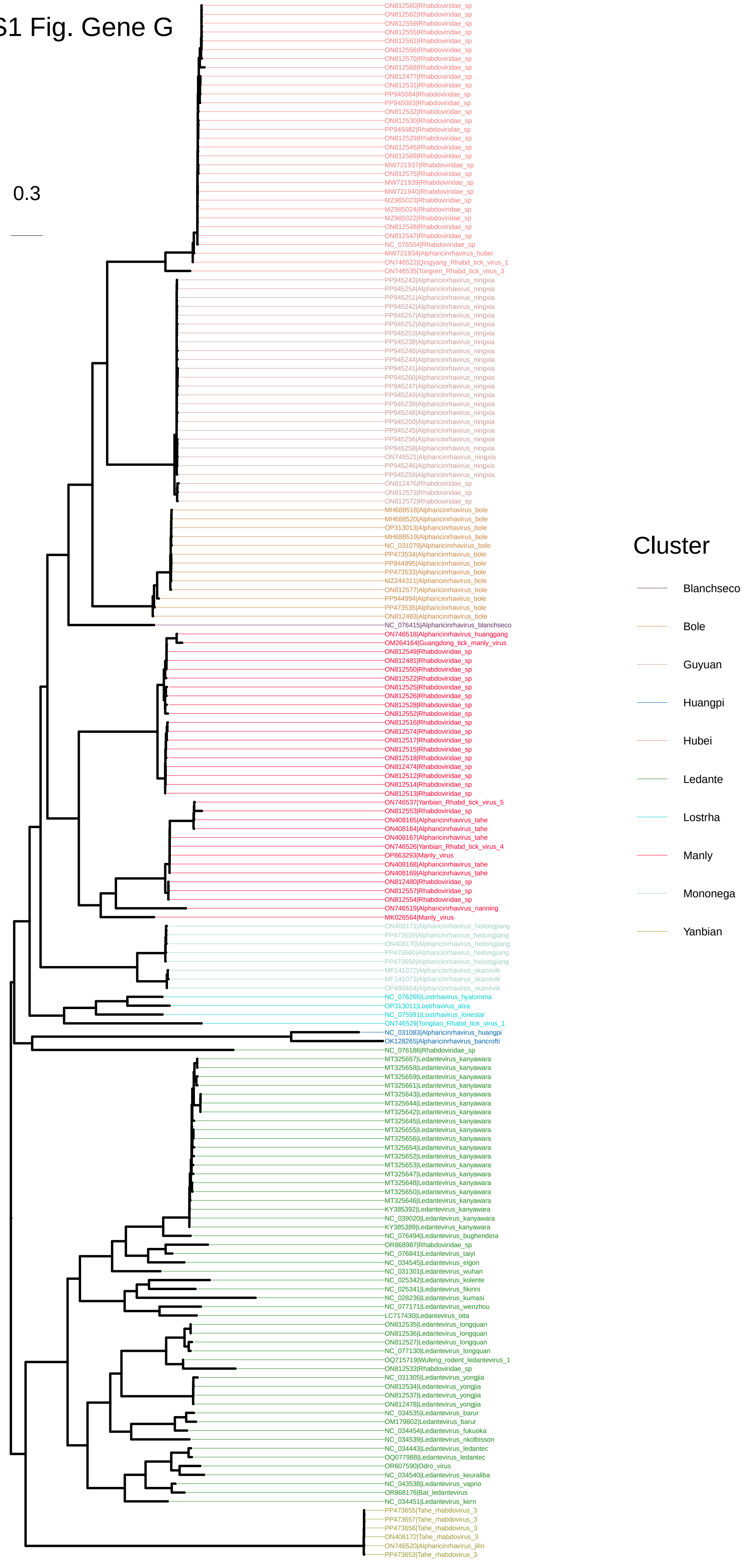

0.3
ON812560|Rhabdoviridae_sp
ON812562|Rhabdoviridae_sp
ON812559|Rhabdoviridae_sp
ON812555|Rhabdoviridae_sp
ON812561|Rhabdoviridae_sp
ON812556|Rhabdoviridae_sp
ON812570|Rhabdoviridae_sp
ON812568|Rhabdoviridae_sp
ON812477|Rhabdoviridae_sp
ON812531|Rhabdoviridae_sp
PP945084|Rhabdoviridae_sp
PP945083|Rhabdoviridae_sp
ON812532|Rhabdoviridae_sp
ON812530|Rhabdoviridae_sp
PP945082|Rhabdoviridae_sp
ON812529|Rhabdoviridae_sp
ON812545|Rhabdoviridae_sp
ON812569|Rhabdoviridae_sp
MW721937|Rhabdoviridae_sp
ON812575|Rhabdoviridae_sp
MW721939|Rhabdoviridae_sp
MW721940|Rhabdoviridae_sp
MZ965023|Rhabdoviridae_sp
MZ965024|Rhabdoviridae_sp
MZ965022|Rhabdoviridae_sp
ON812548|Rhabdoviridae_sp
ON812547|Rhabdoviridae_sp
NC_076504|Rhabdoviridae_sp
MW721934|Alpharicinrhavirus_hubei
ON746522|Qingyang_Rhabd_tick_virus_1
ON746535|Tongren_Rhabd_tick_virus_3
PP945243|Alpharicinrhavirus_ningxia
PP945254|Alpharicinrhavirus_ningxia
PP945251|Alpharicinrhavirus_ningxia
PP945242|Alpharicinrhavirus_ningxia
PP945257|Alpharicinrhavirus_ningxia
PP945252|Alpharicinrhavirus_ningxia
PP945253|Alpharicinrhavirus_ningxia
PP945238|Alpharicinrhavirus_ningxia
PP945240|Alpharicinrhavirus_ningxia
PP945244|Alpharicinrhavirus_ningxia
PP945241|Alpharicinrhavirus_ningxia
PP945260|Alpharicinrhavirus_ningxia
PP945247|Alpharicinrhavirus_ningxia
PP945249|Alpharicinrhavirus_ningxia
PP945239|Alpharicinrhavirus_ningxia
PP945248|Alpharicinrhavirus_ningxia
PP945250|Alpharicinrhavirus_ningxia
PP945245|Alpharicinrhavirus_ningxia
PP945256|Alpharicinrhavirus_ningxia
PP945258|Alpharicinrhavirus_ningxia
ON746521|Alpharicinrhavirus_ningxia
PP945246|Alpharicinrhavirus_ningxia
PP945259|Alpharicinrhavirus_ningxia
ON812476|Rhabdoviridae_sp
ON812573|Rhabdoviridae_sp
ON812572|Rhabdoviridae_sp
MH688518|Alpharicinrhavirus_bole
MH688520|Alpharicinrhavirus_bole
OP313013|Alpharicinrhavirus_bole
MH688519|Alpharicinrhavirus_bole
NC_031079|Alpharicinrhavirus_bole
PP473534|Alpharicinrhavirus_bole
PP944995|Alpharicinrhavirus_bole
PP473533|Alpharicinrhavirus_bole
MZ244311|Alpharicinrhavirus_bole
ON812577|Alpharicinrhavirus_bole
PP944994|Alpharicinrhavirus_bole
PP473535|Alpharicinrhavirus_bole
ON812483|Alpharicinrhavirus_bole
NC_076415|Alpharicinrhavirus_blanchseco
ON746518|Alpharicinrhavirus_huanggang
OM264164|Guangdong_tick_manly_virus
ON812549|Rhabdoviridae_sp
ON812481|Rhabdoviridae_sp
ON812550|Rhabdoviridae_sp
ON812522|Rhabdoviridae_sp
ON812525|Rhabdoviridae_sp
ON812526|Rhabdoviridae_sp
ON812528|Rhabdoviridae_sp
ON812552|Rhabdoviridae_sp
ON812516|Rhabdoviridae_sp
ON812574|Rhabdoviridae_sp
ON812517|Rhabdoviridae_sp
ON812515|Rhabdoviridae_sp
ON812518|Rhabdoviridae_sp
ON812474|Rhabdoviridae_sp
ON812512|Rhabdoviridae_sp
ON812514|Rhabdoviridae_sp
ON812513|Rhabdoviridae_sp
ON746537|Yanbian_Rhabd_tick_virus_5
ON812553|Rhabdoviridae_sp
ON408165|Alpharicinrhavirus_tahe
ON408164|Alpharicinrhavirus_tahe
ON408167|Alpharicinrhavirus_tahe
ON746526|Yanbian_Rhabd_tick_virus_4
OP863293|Manly_virus
ON408168|Alpharicinrhavirus_tahe
ON408169|Alpharicinrhavirus_tahe
ON812480|Rhabdoviridae_sp
ON812557|Rhabdoviridae_sp
ON812554|Rhabdoviridae_sp
ON746519|Alpharicinrhavirus_nanning
MK026564|Manly_virus
ON408171|Alpharicinrhavirus_heilongjiang
PP473659|Alpharicinrhavirus_heilongjiang
ON408170|Alpharicinrhavirus_heilongjiang
PP473660|Alpharicinrhavirus_heilongjiang
PP473658|Alpharicinrhavirus_heilongjiang
MF141072|Alpharicinrhavirus_skanevik
MF141073|Alpharicinrhavirus_skanevik
OP480464|Alpharicinrhavirus_skanevik
NC_076266|Lostrhavirus_hyalomma
OP313011|Lostrhavirus_alxa
NC_075991|Lostrhavirus_lonestar
ON746529|Tongliao_Rhabd_tick_virus_1
NC_031083|Alpharicinrhavirus_huangpi
OK128265|Alpharicinrhavirus_bancrofti
NC_076186|Rhabdoviridae_sp
MT325657|Ledantevirus_kanyawara
MT325658|Ledantevirus_kanyawara
MT325659|Ledantevirus_kanyawara
MT325661|Ledantevirus_kanyawara
MT325643|Ledantevirus_kanyawara
MT325644|Ledantevirus_kanyawara
MT325642|Ledantevirus_kanyawara
MT325645|Ledantevirus_kanyawara
MT325655|Ledantevirus_kanyawara
MT325656|Ledantevirus_kanyawara
MT325654|Ledantevirus_kanyawara
MT325652|Ledantevirus_kanyawara
MT325653|Ledantevirus_kanyawara
MT325647|Ledantevirus_kanyawara
MT325648|Ledantevirus_kanyawara
MT325650|Ledantevirus_kanyawara
MT325646|Ledantevirus_kanyawara
KY385392|Ledantevirus_kanyawara
NC_039020|Ledantevirus_kanyawara
KY385389|Ledantevirus_kanyawara
NC_076494|Ledantevirus_bughendera
OR868987|Rhabdoviridae_sp
NC_076841|Ledantevirus_taiyi
NC_034545|Ledantevirus_elgon
NC_031301|Ledantevirus_wuhan
NC_025342|Ledantevirus_kolente
NC_025341|Ledantevirus_fikirini
NC_028236|Ledantevirus_kumasi
NC_077171|Ledantevirus_wenzhou
LC717430|Ledantevirus_oita
ON812535|Ledantevirus_longquan
ON812536|Ledantevirus_longquan
ON812527|Ledantevirus_longquan
NC_077130|Ledantevirus_longquan
OQ715719|Wufeng_rodent_ledantevirus_1
ON812533|Rhabdoviridae_sp
NC_031305|Ledantevirus_yongjia
ON812534|Ledantevirus_yongjia
ON812537|Ledantevirus_yongjia
ON812478|Ledantevirus_yongjia
NC_034535|Ledantevirus_barur
OM179802|Ledantevirus_barur
NC_034454|Ledantevirus_fukuoka
NC_034539|Ledantevirus_nkolbisson
NC_034443|Ledantevirus_ledantec
OQ077988|Ledantevirus_ledantec
OR607590|Odro_virus
NC_034540|Ledantevirus_keuraliba
NC_043538|Ledantevirus_vaprio
OR868176|Bat_ledantevirus
NC_034451|Ledantevirus_kern
PP473655|Tahe_rhabdovirus_3
PP473657|Tahe_rhabdovirus_3
PP473656|Tahe_rhabdovirus_3
ON408172|Tahe_rhabdovirus_3
ON746520|Alpharicinrhavirus_jilin
PP473653|Tahe_rhabdovirus_3
Cluster
Blanchseco
Bole
Guyuan
Huangpi
Hubei
Ledante
Lostrha
Manly
Mononega
Yanbian

S1 Fig. Gene L

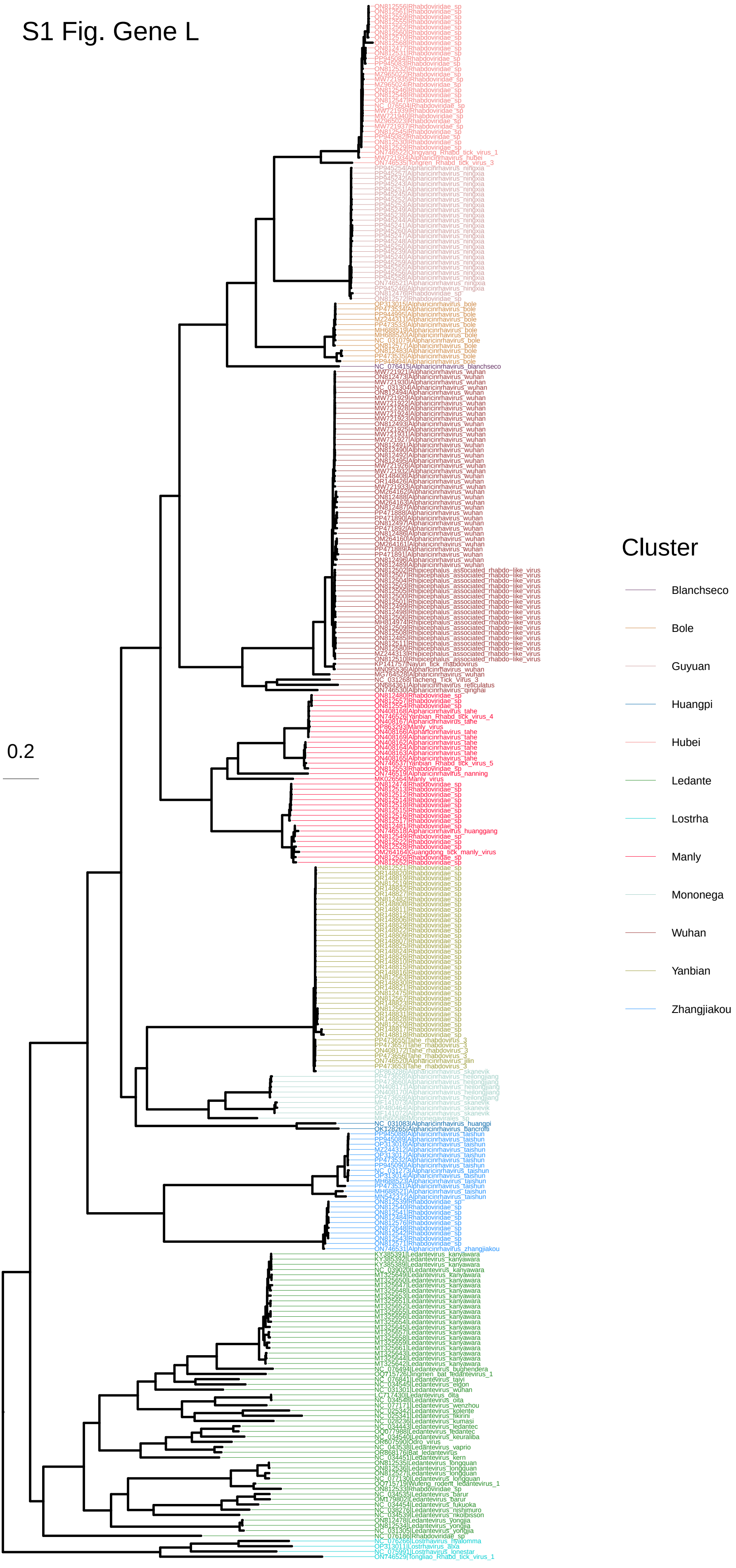
0.2
Cluster
Blanchseco
Bole
Guyuan
Huangpi
Hubei
Ledante
Lostrha
Manly
Mononega
Wuhan
Yanbian
Zhangjiakou

S1 Fig. Gene M

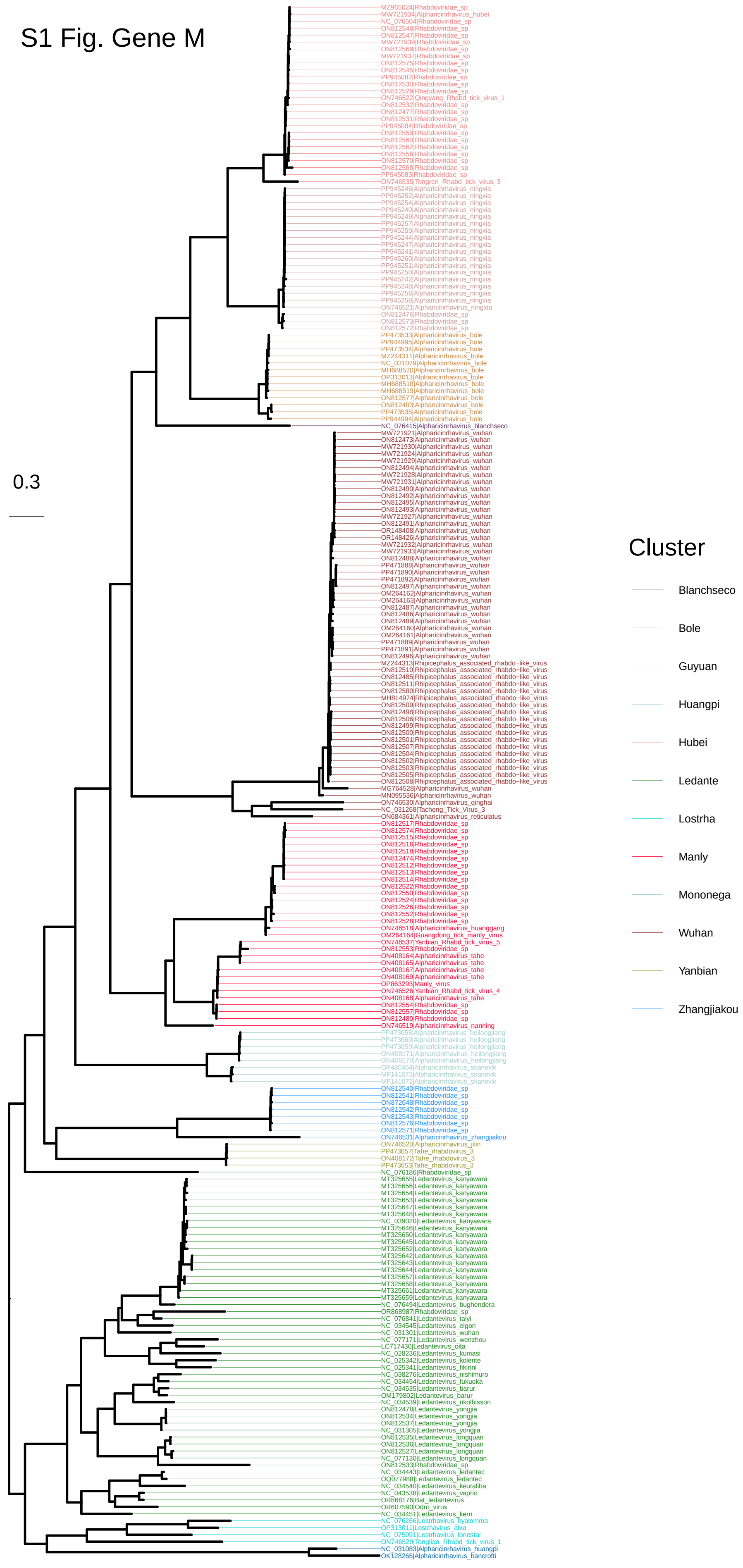

0.3
MZ965024|Rhabdoviridae_sp
MW721934|Alpharicinrhavirus_hubei
NC_076504|Rhabdoviridae_sp
ON812548|Rhabdoviridae_sp
ON812547|Rhabdoviridae_sp
MW721939|Rhabdoviridae_sp
ON812569|Rhabdoviridae_sp
MW721937|Rhabdoviridae_sp
ON812575|Rhabdoviridae_sp
ON812545|Rhabdoviridae_sp
PP945082|Rhabdoviridae_sp
ON812530|Rhabdoviridae_sp
ON812529|Rhabdoviridae_sp
ON746522|Qingyang_Rhabd_tick_virus_1
ON812532|Rhabdoviridae_sp
ON812477|Rhabdoviridae_sp
ON812531|Rhabdoviridae_sp
PP945084|Rhabdoviridae_sp
ON812559|Rhabdoviridae_sp
ON812560|Rhabdoviridae_sp
ON812562|Rhabdoviridae_sp
ON812556|Rhabdoviridae_sp
ON812570|Rhabdoviridae_sp
ON812568|Rhabdoviridae_sp
PP945083|Rhabdoviridae_sp
ON746535|Tongren_Rhabd_tick_virus_3
PP945246|Alpharicinrhavirus_ningxia
PP945252|Alpharicinrhavirus_ningxia
PP945254|Alpharicinrhavirus_ningxia
PP945240|Alpharicinrhavirus_ningxia
PP945249|Alpharicinrhavirus_ningxia
PP945257|Alpharicinrhavirus_ningxia
PP945259|Alpharicinrhavirus_ningxia
PP945244|Alpharicinrhavirus_ningxia
PP945247|Alpharicinrhavirus_ningxia
PP945241|Alpharicinrhavirus_ningxia
PP945260|Alpharicinrhavirus_ningxia
PP945251|Alpharicinrhavirus_ningxia
PP945250|Alpharicinrhavirus_ningxia
PP945242|Alpharicinrhavirus_ningxia
PP945248|Alpharicinrhavirus_ningxia
PP945256|Alpharicinrhavirus_ningxia
PP945258|Alpharicinrhavirus_ningxia
ON746521|Alpharicinrhavirus_ningxia
ON812476|Rhabdoviridae_sp
ON812573|Rhabdoviridae_sp
ON812572|Rhabdoviridae_sp
PP473533|Alpharicinrhavirus_bole
PP944995|Alpharicinrhavirus_bole
PP473534|Alpharicinrhavirus_bole
MZ244311|Alpharicinrhavirus_bole
NC_031079|Alpharicinrhavirus_bole
MH688520|Alpharicinrhavirus_bole
OP313013|Alpharicinrhavirus_bole
MH688518|Alpharicinrhavirus_bole
MH688519|Alpharicinrhavirus_bole
ON812577|Alpharicinrhavirus_bole
ON812483|Alpharicinrhavirus_bole
PP473535|Alpharicinrhavirus_bole
PP944994|Alpharicinrhavirus_bole
NC_076415|Alpharicinrhavirus_blanchseco
MW721921|Alpharicinrhavirus_wuhan
ON812473|Alpharicinrhavirus_wuhan
MW721930|Alpharicinrhavirus_wuhan
MW721924|Alpharicinrhavirus_wuhan
MW721929|Alpharicinrhavirus_wuhan
ON812494|Alpharicinrhavirus_wuhan
MW721928|Alpharicinrhavirus_wuhan
MW721931|Alpharicinrhavirus_wuhan
ON812490|Alpharicinrhavirus_wuhan
ON812492|Alpharicinrhavirus_wuhan
ON812495|Alpharicinrhavirus_wuhan
ON812493|Alpharicinrhavirus_wuhan
MW721927|Alpharicinrhavirus_wuhan
ON812491|Alpharicinrhavirus_wuhan
OR148408|Alpharicinrhavirus_wuhan
OR148426|Alpharicinrhavirus_wuhan
MW721932|Alpharicinrhavirus_wuhan
MW721933|Alpharicinrhavirus_wuhan
ON812488|Alpharicinrhavirus_wuhan
PP471888|Alpharicinrhavirus_wuhan
PP471890|Alpharicinrhavirus_wuhan
PP471892|Alpharicinrhavirus_wuhan
ON812497|Alpharicinrhavirus_wuhan
OM264162|Alpharicinrhavirus_wuhan
OM264163|Alpharicinrhavirus_wuhan
ON812487|Alpharicinrhavirus_wuhan
ON812486|Alpharicinrhavirus_wuhan
ON812489|Alpharicinrhavirus_wuhan
OM264160|Alpharicinrhavirus_wuhan
OM264161|Alpharicinrhavirus_wuhan
PP471889|Alpharicinrhavirus_wuhan
PP471891|Alpharicinrhavirus_wuhan
ON812496|Alpharicinrhavirus_wuhan
MZ244313|Rhipicephalus_associated_rhabdo-like_virus
ON812510|Rhipicephalus_associated_rhabdo-like_virus
ON812485|Rhipicephalus_associated_rhabdo-like_virus
ON812511|Rhipicephalus_associated_rhabdo-like_virus
ON812580|Rhipicephalus_associated_rhabdo-like_virus
MH814974|Rhipicephalus_associated_rhabdo-like_virus
ON812509|Rhipicephalus_associated_rhabdo-like_virus
ON812498|Rhipicephalus_associated_rhabdo-like_virus
ON812506|Rhipicephalus_associated_rhabdo-like_virus
ON812499|Rhipicephalus_associated_rhabdo-like_virus
ON812500|Rhipicephalus_associated_rhabdo-like_virus
ON812501|Rhipicephalus_associated_rhabdo-like_virus
ON812507|Rhipicephalus_associated_rhabdo-like_virus
ON812504|Rhipicephalus_associated_rhabdo-like_virus
ON812502|Rhipicephalus_associated_rhabdo-like_virus
ON812503|Rhipicephalus_associated_rhabdo-like_virus
ON812505|Rhipicephalus_associated_rhabdo-like_virus
ON812508|Rhipicephalus_associated_rhabdo-like_virus
MG764528|Alpharicinrhavirus_wuhan
MN095536|Alpharicinrhavirus_wuhan
ON746530|Alpharicinrhavirus_qinghai
NC_031268|Tacheng_Tick_Virus_3
ON684361|Alpharicinrhavirus_reticulatus
ON812517|Rhabdoviridae_sp
ON812574|Rhabdoviridae_sp
ON812515|Rhabdoviridae_sp
ON812516|Rhabdoviridae_sp
ON812518|Rhabdoviridae_sp
ON812474|Rhabdoviridae_sp
ON812512|Rhabdoviridae_sp
ON812513|Rhabdoviridae_sp
ON812514|Rhabdoviridae_sp
ON812522|Rhabdoviridae_sp
ON812550|Rhabdoviridae_sp
ON812524|Rhabdoviridae_sp
ON812526|Rhabdoviridae_sp
ON812552|Rhabdoviridae_sp
ON812528|Rhabdoviridae_sp
ON746518|Alpharicinrhavirus_huanggang
OM264164|Guangdong_tick_manly_virus
ON746537|Yanbian_Rhabd_tick_virus_5
ON812553|Rhabdoviridae_sp
ON408164|Alpharicinrhavirus_tahe
ON408165|Alpharicinrhavirus_tahe
ON408167|Alpharicinrhavirus_tahe
ON408169|Alpharicinrhavirus_tahe
OP863293|Manly_virus
ON746526|Yanbian_Rhabd_tick_virus_4
ON408168|Alpharicinrhavirus_tahe
ON812554|Rhabdoviridae_sp
ON812557|Rhabdoviridae_sp
ON812480|Rhabdoviridae_sp
ON746519|Alpharicinrhavirus_nanning
PP473658|Alpharicinrhavirus_heilongjiang
PP473660|Alpharicinrhavirus_heilongjiang
PP473659|Alpharicinrhavirus_heilongjiang
ON408171|Alpharicinrhavirus_heilongjiang
ON408170|Alpharicinrhavirus_heilongjiang
OP480464|Alpharicinrhavirus_skanevik
MF141073|Alpharicinrhavirus_skanevik
MF141072|Alpharicinrhavirus_skanevik
ON812540|Rhabdoviridae_sp
ON812541|Rhabdoviridae_sp
ON872648|Rhabdoviridae_sp
ON812542|Rhabdoviridae_sp
ON812543|Rhabdoviridae_sp
ON812576|Rhabdoviridae_sp
ON812571|Rhabdoviridae_sp
ON746531|Alpharicinrhavirus_zhangjiakou
ON746520|Alpharicinrhavirus_jilin
PP473657|Tahe_rhabdovirus_3
ON408172|Tahe_rhabdovirus_3
PP473653|Tahe_rhabdovirus_3
NC_076186|Rhabdoviridae_sp
MT325655|Ledantevirus_kanyawara
MT325656|Ledantevirus_kanyawara
MT325654|Ledantevirus_kanyawara
MT325653|Ledantevirus_kanyawara
MT325647|Ledantevirus_kanyawara
MT325648|Ledantevirus_kanyawara
NC_039020|Ledantevirus_kanyawara
MT325646|Ledantevirus_kanyawara
MT325650|Ledantevirus_kanyawara
MT325645|Ledantevirus_kanyawara
MT325652|Ledantevirus_kanyawara
MT325642|Ledantevirus_kanyawara
MT325643|Ledantevirus_kanyawara
MT325644|Ledantevirus_kanyawara
MT325657|Ledantevirus_kanyawara
MT325658|Ledantevirus_kanyawara
MT325661|Ledantevirus_kanyawara
MT325659|Ledantevirus_kanyawara
NC_076494|Ledantevirus_bughendera
OR868987|Rhabdoviridae_sp
NC_076841|Ledantevirus_taiyi
NC_034545|Ledantevirus_elgon
NC_031301|Ledantevirus_wuhan
NC_077171|Ledantevirus_wenzhou
LC717430|Ledantevirus_oita
NC_028236|Ledantevirus_kumasi
NC_025342|Ledantevirus_kolente
NC_025341|Ledantevirus_fikirini
NC_038276|Ledantevirus_nishimuro
NC_034454|Ledantevirus_fukuoka
NC_034535|Ledantevirus_barur
OM179802|Ledantevirus_barur
NC_034539|Ledantevirus_nkolbisson
ON812478|Ledantevirus_yongjia
ON812534|Ledantevirus_yongjia
ON812537|Ledantevirus_yongjia
NC_031305|Ledantevirus_yongjia
ON812535|Ledantevirus_longquan
ON812536|Ledantevirus_longquan
ON812527|Ledantevirus_longquan
NC_077130|Ledantevirus_longquan
ON812533|Rhabdoviridae_sp
NC_034443|Ledantevirus_ledantec
OQ077988|Ledantevirus_ledantec
NC_034540|Ledantevirus_keuraliba
NC_043538|Ledantevirus_vaprio
OR868176|Bat_ledantevirus
OR607590|Odro_virus
NC_034451|Ledantevirus_kern
NC_076266|Lostrhavirus_hyalomma
OP313011|Lostrhavirus_alxa
NC_075991|Lostrhavirus_lonestar
ON746529|Tongliao_Rhabd_tick_virus_1
NC_031083|Alpharicinrhavirus_huangpi
OK128265|Alpharicinrhavirus_bancrofti
Cluster
Blanchseco
Bole
Guyuan
Huangpi
Hubei
Ledante
Lostrha
Manly
Mononega
Wuhan
Yanbian
Zhangjiakou

S1 Fig. Gene N

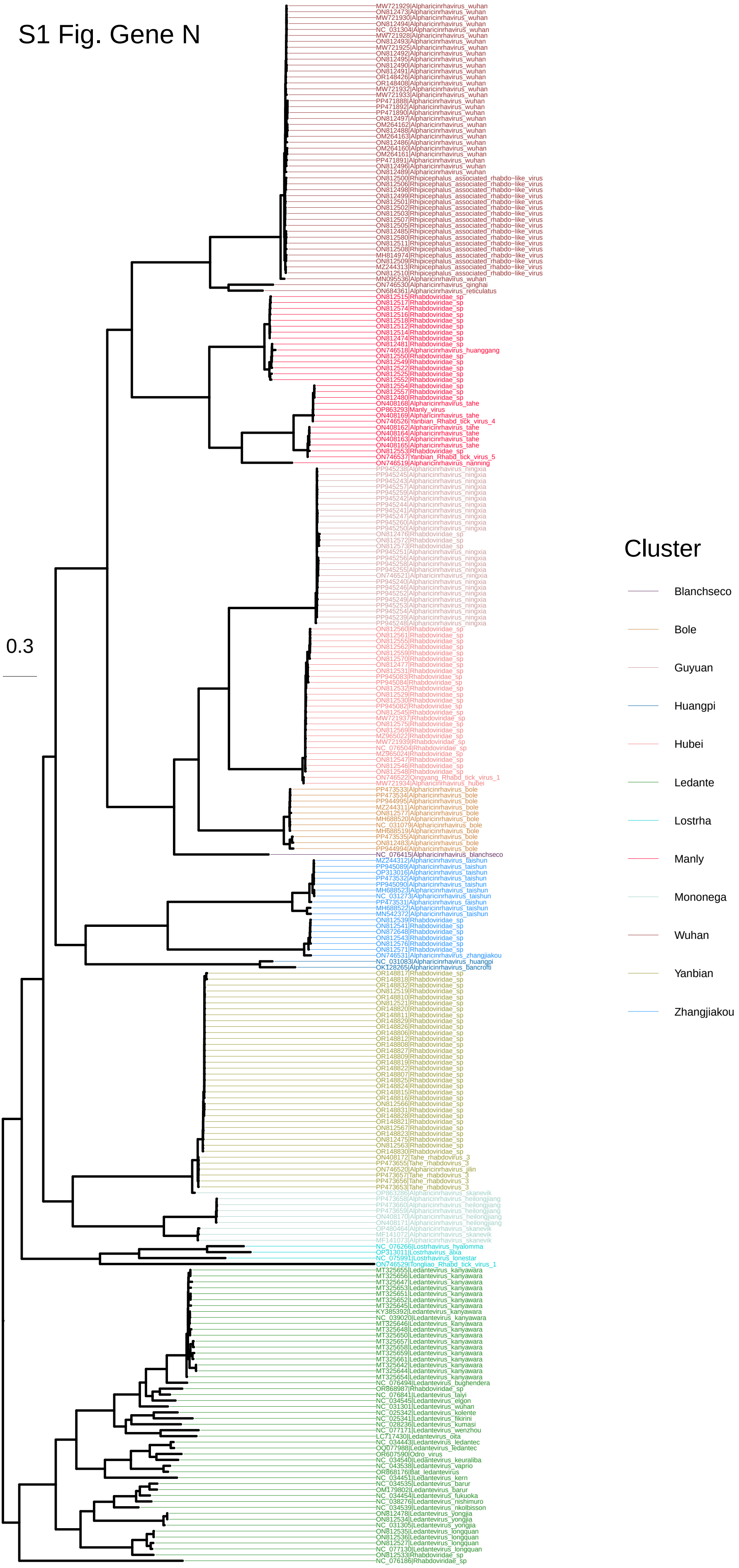

0.3
MW721929|Alpharicinrhavirus_wuhan
ON812473|Alpharicinrhavirus_wuhan
MW721930|Alpharicinrhavirus_wuhan
ON812494|Alpharicinrhavirus_wuhan
NC_031304|Alpharicinrhavirus_wuhan
MW721928|Alpharicinrhavirus_wuhan
ON812493|Alpharicinrhavirus_wuhan
MW721925|Alpharicinrhavirus_wuhan
ON812492|Alpharicinrhavirus_wuhan
ON812495|Alpharicinrhavirus_wuhan
ON812490|Alpharicinrhavirus_wuhan
ON812491|Alpharicinrhavirus_wuhan
OR148426|Alpharicinrhavirus_wuhan
OR148408|Alpharicinrhavirus_wuhan
MW721932|Alpharicinrhavirus_wuhan
MW721933|Alpharicinrhavirus_wuhan
PP471888|Alpharicinrhavirus_wuhan
PP471892|Alpharicinrhavirus_wuhan
PP471890|Alpharicinrhavirus_wuhan
ON812497|Alpharicinrhavirus_wuhan
OM264162|Alpharicinrhavirus_wuhan
ON812488|Alpharicinrhavirus_wuhan
OM264163|Alpharicinrhavirus_wuhan
ON812486|Alpharicinrhavirus_wuhan
OM264160|Alpharicinrhavirus_wuhan
OM264161|Alpharicinrhavirus_wuhan
PP471891|Alpharicinrhavirus_wuhan
ON812496|Alpharicinrhavirus_wuhan
ON812489|Alpharicinrhavirus_wuhan
ON812500|Rhipicephalus_associated_rhabdo−like_virus
ON812506|Rhipicephalus_associated_rhabdo−like_virus
ON812498|Rhipicephalus_associated_rhabdo−like_virus
ON812499|Rhipicephalus_associated_rhabdo−like_virus
ON812501|Rhipicephalus_associated_rhabdo−like_virus
ON812502|Rhipicephalus_associated_rhabdo−like_virus
ON812503|Rhipicephalus_associated_rhabdo−like_virus
ON812507|Rhipicephalus_associated_rhabdo−like_virus
ON812505|Rhipicephalus_associated_rhabdo−like_virus
ON812485|Rhipicephalus_associated_rhabdo−like_virus
ON812580|Rhipicephalus_associated_rhabdo−like_virus
ON812511|Rhipicephalus_associated_rhabdo−like_virus
ON812508|Rhipicephalus_associated_rhabdo−like_virus
MH814974|Rhipicephalus_associated_rhabdo−like_virus
ON812509|Rhipicephalus_associated_rhabdo−like_virus
MZ244313|Rhipicephalus_associated_rhabdo−like_virus
ON812510|Rhipicephalus_associated_rhabdo−like_virus
MN095536|Alpharicinrhavirus_wuhan
ON746530|Alpharicinrhavirus_qinghai
ON684361|Alpharicinrhavirus_reticulatus
ON812515|Rhabdoviridae_sp
ON812517|Rhabdoviridae_sp
ON812574|Rhabdoviridae_sp
ON812516|Rhabdoviridae_sp
ON812518|Rhabdoviridae_sp
ON812512|Rhabdoviridae_sp
ON812514|Rhabdoviridae_sp
ON812474|Rhabdoviridae_sp
ON812481|Rhabdoviridae_sp
ON746518|Alpharicinrhavirus_huanggang
ON812550|Rhabdoviridae_sp
ON812549|Rhabdoviridae_sp
ON812522|Rhabdoviridae_sp
ON812525|Rhabdoviridae_sp
ON812552|Rhabdoviridae_sp
ON812554|Rhabdoviridae_sp
ON812557|Rhabdoviridae_sp
ON812480|Rhabdoviridae_sp
ON408168|Alpharicinrhavirus_tahe
OP863293|Manly_virus
ON408169|Alpharicinrhavirus_tahe
ON746526|Yanbian_Rhabd_tick_virus_4
ON408162|Alpharicinrhavirus_tahe
ON408164|Alpharicinrhavirus_tahe
ON408163|Alpharicinrhavirus_tahe
ON408165|Alpharicinrhavirus_tahe
ON812553|Rhabdoviridae_sp
ON746537|Yanbian_Rhabd_tick_virus_5
ON746519|Alpharicinrhavirus_nanning
PP945238|Alpharicinrhavirus_ningxia
PP945245|Alpharicinrhavirus_ningxia
PP945243|Alpharicinrhavirus_ningxia
PP945257|Alpharicinrhavirus_ningxia
PP945259|Alpharicinrhavirus_ningxia
PP945242|Alpharicinrhavirus_ningxia
PP945244|Alpharicinrhavirus_ningxia
PP945241|Alpharicinrhavirus_ningxia
PP945247|Alpharicinrhavirus_ningxia
PP945260|Alpharicinrhavirus_ningxia
PP945250|Alpharicinrhavirus_ningxia
ON812476|Rhabdoviridae_sp
ON812572|Rhabdoviridae_sp
ON812573|Rhabdoviridae_sp
PP945251|Alpharicinrhavirus_ningxia
PP945256|Alpharicinrhavirus_ningxia
PP945258|Alpharicinrhavirus_ningxia
PP945255|Alpharicinrhavirus_ningxia
ON746521|Alpharicinrhavirus_ningxia
PP945240|Alpharicinrhavirus_ningxia
PP945246|Alpharicinrhavirus_ningxia
PP945252|Alpharicinrhavirus_ningxia
PP945249|Alpharicinrhavirus_ningxia
PP945253|Alpharicinrhavirus_ningxia
PP945254|Alpharicinrhavirus_ningxia
PP945239|Alpharicinrhavirus_ningxia
PP945248|Alpharicinrhavirus_ningxia
ON812560|Rhabdoviridae_sp
ON812561|Rhabdoviridae_sp
ON812555|Rhabdoviridae_sp
ON812562|Rhabdoviridae_sp
ON812559|Rhabdoviridae_sp
ON812570|Rhabdoviridae_sp
ON812477|Rhabdoviridae_sp
ON812531|Rhabdoviridae_sp
PP945083|Rhabdoviridae_sp
PP945084|Rhabdoviridae_sp
ON812532|Rhabdoviridae_sp
ON812529|Rhabdoviridae_sp
ON812530|Rhabdoviridae_sp
PP945082|Rhabdoviridae_sp
ON812545|Rhabdoviridae_sp
MW721937|Rhabdoviridae_sp
ON812575|Rhabdoviridae_sp
ON812569|Rhabdoviridae_sp
MZ965022|Rhabdoviridae_sp
MW721939|Rhabdoviridae_sp
NC_076504|Rhabdoviridae_sp
MZ965024|Rhabdoviridae_sp
ON812547|Rhabdoviridae_sp
ON812546|Rhabdoviridae_sp
ON812548|Rhabdoviridae_sp
ON746522|Qingyang_Rhabd_tick_virus_1
MW721934|Alpharicinrhavirus_hubei
PP473533|Alpharicinrhavirus_bole
PP473534|Alpharicinrhavirus_bole
PP944995|Alpharicinrhavirus_bole
MZ244311|Alpharicinrhavirus_bole
ON812577|Alpharicinrhavirus_bole
MH688520|Alpharicinrhavirus_bole
NC_031079|Alpharicinrhavirus_bole
MH688519|Alpharicinrhavirus_bole
PP473535|Alpharicinrhavirus_bole
ON812483|Alpharicinrhavirus_bole
PP944994|Alpharicinrhavirus_bole
NC_076415|Alpharicinrhavirus_blanchseco
MZ244312|Alpharicinrhavirus_taishun
PP945089|Alpharicinrhavirus_taishun
OP313016|Alpharicinrhavirus_taishun
PP473532|Alpharicinrhavirus_taishun
PP945090|Alpharicinrhavirus_taishun
MH688523|Alpharicinrhavirus_taishun
NC_031273|Alpharicinrhavirus_taishun
PP473531|Alpharicinrhavirus_taishun
MH688522|Alpharicinrhavirus_taishun
MN542372|Alpharicinrhavirus_taishun
ON812539|Rhabdoviridae_sp
ON812541|Rhabdoviridae_sp
ON872648|Rhabdoviridae_sp
ON812543|Rhabdoviridae_sp
ON812576|Rhabdoviridae_sp
ON812571|Rhabdoviridae_sp
ON746531|Alpharicinrhavirus_zhangjiakou
NC_031083|Alpharicinrhavirus_huangpi
OK128265|Alpharicinrhavirus_bancrofti
OR148817|Rhabdoviridae_sp
OR148818|Rhabdoviridae_sp
OR148832|Rhabdoviridae_sp
ON812519|Rhabdoviridae_sp
OR148810|Rhabdoviridae_sp
ON812521|Rhabdoviridae_sp
OR148820|Rhabdoviridae_sp
OR148811|Rhabdoviridae_sp
OR148829|Rhabdoviridae_sp
OR148826|Rhabdoviridae_sp
OR148806|Rhabdoviridae_sp
OR148812|Rhabdoviridae_sp
OR148808|Rhabdoviridae_sp
OR148827|Rhabdoviridae_sp
OR148809|Rhabdoviridae_sp
OR148819|Rhabdoviridae_sp
OR148822|Rhabdoviridae_sp
OR148807|Rhabdoviridae_sp
OR148825|Rhabdoviridae_sp
OR148824|Rhabdoviridae_sp
OR148815|Rhabdoviridae_sp
OR148816|Rhabdoviridae_sp
ON812566|Rhabdoviridae_sp
OR148831|Rhabdoviridae_sp
OR148828|Rhabdoviridae_sp
OR148821|Rhabdoviridae_sp
ON812567|Rhabdoviridae_sp
OR148823|Rhabdoviridae_sp
ON812475|Rhabdoviridae_sp
ON812563|Rhabdoviridae_sp
OR148830|Rhabdoviridae_sp
ON408172|Tahe_rhabdovirus_3
PP473655|Tahe_rhabdovirus_3
ON746520|Alpharicinrhavirus_jilin
PP473657|Tahe_rhabdovirus_3
PP473656|Tahe_rhabdovirus_3
PP473653|Tahe_rhabdovirus_3
OP863286|Alpharicinrhavirus_skanevik
PP473658|Alpharicinrhavirus_heilongjiang
PP473660|Alpharicinrhavirus_heilongjiang
PP473659|Alpharicinrhavirus_heilongjiang
ON408170|Alpharicinrhavirus_heilongjiang
ON408171|Alpharicinrhavirus_heilongjiang
OP480464|Alpharicinrhavirus_skanevik
MF141072|Alpharicinrhavirus_skanevik
MF141073|Alpharicinrhavirus_skanevik
NC_076266|Lostrhavirus_hyalomma
OP313011|Lostrhavirus_alxa
NC_075991|Lostrhavirus_lonestar
ON746529|Tongliao_Rhabd_tick_virus_1
MT325655|Ledantevirus_kanyawara
MT325656|Ledantevirus_kanyawara
MT325647|Ledantevirus_kanyawara
MT325653|Ledantevirus_kanyawara
MT325651|Ledantevirus_kanyawara
MT325652|Ledantevirus_kanyawara
MT325645|Ledantevirus_kanyawara
KY385392|Ledantevirus_kanyawara
NC_039020|Ledantevirus_kanyawara
MT325646|Ledantevirus_kanyawara
MT325648|Ledantevirus_kanyawara
MT325650|Ledantevirus_kanyawara
MT325657|Ledantevirus_kanyawara
MT325658|Ledantevirus_kanyawara
MT325659|Ledantevirus_kanyawara
MT325661|Ledantevirus_kanyawara
MT325642|Ledantevirus_kanyawara
MT325644|Ledantevirus_kanyawara
MT325654|Ledantevirus_kanyawara
NC_076494|Ledantevirus_bughendera
OR868987|Rhabdoviridae_sp
NC_076841|Ledantevirus_taiyi
NC_034545|Ledantevirus_elgon
NC_031301|Ledantevirus_wuhan
NC_025342|Ledantevirus_kolente
NC_025341|Ledantevirus_fikirini
NC_028236|Ledantevirus_kumasi
NC_077171|Ledantevirus_wenzhou
LC717430|Ledantevirus_oita
NC_034443|Ledantevirus_ledantec
OQ077988|Ledantevirus_ledantec
OR607590|Odro_virus
NC_034540|Ledantevirus_keuraliba
NC_043538|Ledantevirus_vaprio
OR868176|Bat_ledantevirus
NC_034451|Ledantevirus_kern
NC_034535|Ledantevirus_barur
OM179802|Ledantevirus_barur
NC_034454|Ledantevirus_fukuoka
NC_038276|Ledantevirus_nishimuro
NC_034539|Ledantevirus_nkolbisson
ON812478|Ledantevirus_yongjia
ON812534|Ledantevirus_yongjia
NC_031305|Ledantevirus_yongjia
ON812535|Ledantevirus_longquan
ON812536|Ledantevirus_longquan
ON812527|Ledantevirus_longquan
NC_077130|Ledantevirus_longquan
ON812533|Rhabdoviridae_sp
NC_076186|Rhabdoviridae_sp
Cluster
Blanchseco
Bole
Guyuan
Huangpi
Hubei
Ledante
Lostrha
Manly
Mononega
Wuhan
Yanbian
Zhangjiakou

S1 Fig. Gene P

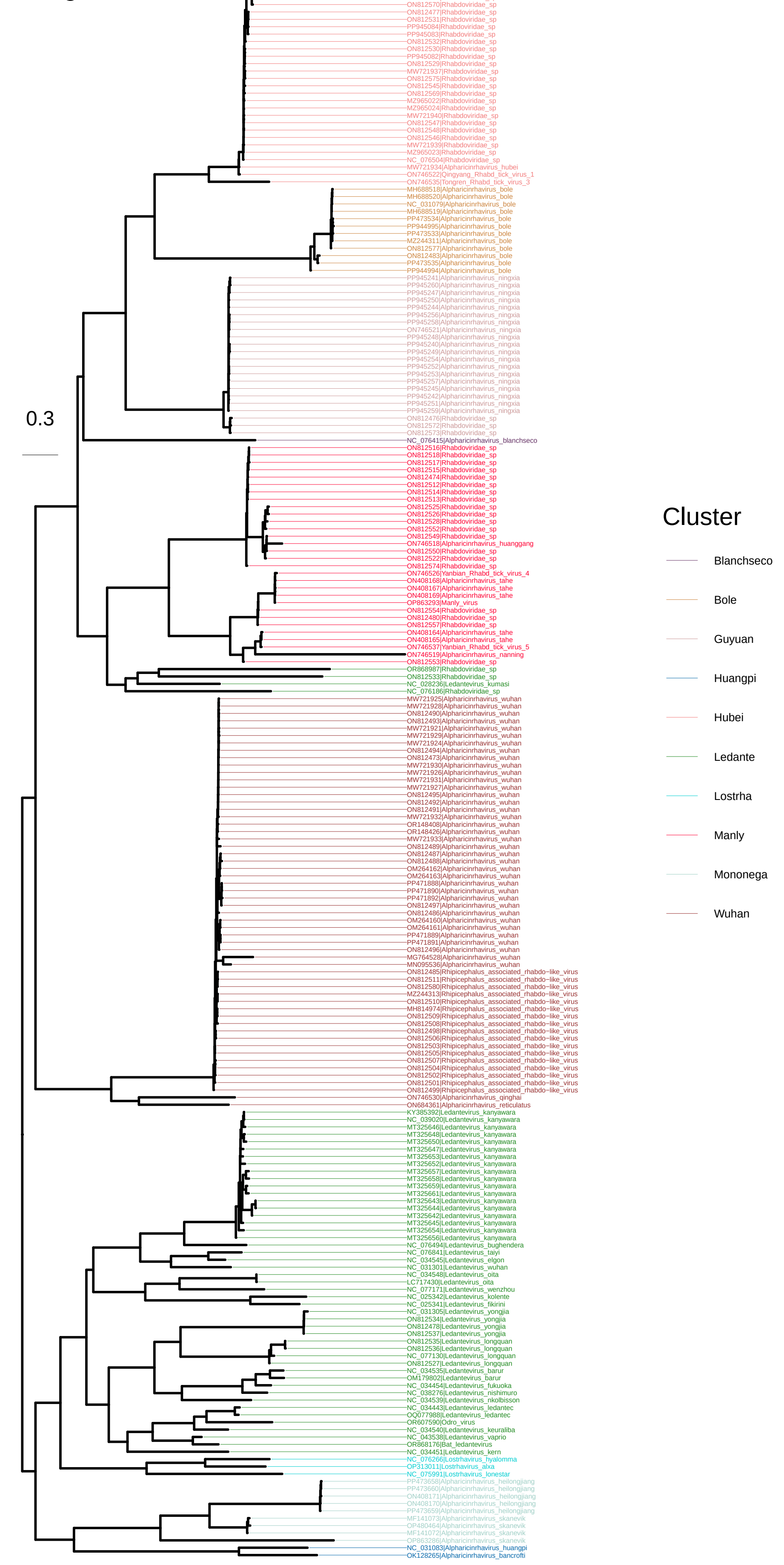
0.3
ON812555|Rhabdoviridae_sp
ON812556|Rhabdoviridae_sp
ON812561|Rhabdoviridae_sp
ON812560|Rhabdoviridae_sp
ON812562|Rhabdoviridae_sp
ON812559|Rhabdoviridae_sp
ON812570|Rhabdoviridae_sp
ON812477|Rhabdoviridae_sp
ON812531|Rhabdoviridae_sp
PP945084|Rhabdoviridae_sp
PP945083|Rhabdoviridae_sp
ON812532|Rhabdoviridae_sp
ON812530|Rhabdoviridae_sp
PP945082|Rhabdoviridae_sp
ON812529|Rhabdoviridae_sp
MW721937|Rhabdoviridae_sp
ON812575|Rhabdoviridae_sp
ON812545|Rhabdoviridae_sp
ON812569|Rhabdoviridae_sp
MZ965022|Rhabdoviridae_sp
MZ965024|Rhabdoviridae_sp
MW721940|Rhabdoviridae_sp
ON812547|Rhabdoviridae_sp
ON812548|Rhabdoviridae_sp
ON812546|Rhabdoviridae_sp
MW721939|Rhabdoviridae_sp
MZ965023|Rhabdoviridae_sp
NC_076504|Rhabdoviridae_sp
MW721934|Alpharicinrhavirus_hubei
ON746522|Qingyang_Rhabd_tick_virus_1
ON746535|Tongren_Rhabd_tick_virus_3
MH688518|Alpharicinrhavirus_bole
MH688520|Alpharicinrhavirus_bole
NC_031079|Alpharicinrhavirus_bole
MH688519|Alpharicinrhavirus_bole
PP473534|Alpharicinrhavirus_bole
PP944995|Alpharicinrhavirus_bole
PP473533|Alpharicinrhavirus_bole
MZ244311|Alpharicinrhavirus_bole
ON812577|Alpharicinrhavirus_bole
ON812483|Alpharicinrhavirus_bole
PP473535|Alpharicinrhavirus_bole
PP944994|Alpharicinrhavirus_bole
PP945241|Alpharicinrhavirus_ningxia
PP945260|Alpharicinrhavirus_ningxia
PP945247|Alpharicinrhavirus_ningxia
PP945250|Alpharicinrhavirus_ningxia
PP945244|Alpharicinrhavirus_ningxia
PP945256|Alpharicinrhavirus_ningxia
PP945258|Alpharicinrhavirus_ningxia
ON746521|Alpharicinrhavirus_ningxia
PP945248|Alpharicinrhavirus_ningxia
PP945240|Alpharicinrhavirus_ningxia
PP945249|Alpharicinrhavirus_ningxia
PP945254|Alpharicinrhavirus_ningxia
PP945252|Alpharicinrhavirus_ningxia
PP945253|Alpharicinrhavirus_ningxia
PP945257|Alpharicinrhavirus_ningxia
PP945245|Alpharicinrhavirus_ningxia
PP945242|Alpharicinrhavirus_ningxia
PP945251|Alpharicinrhavirus_ningxia
PP945259|Alpharicinrhavirus_ningxia
ON812476|Rhabdoviridae_sp
ON812572|Rhabdoviridae_sp
ON812573|Rhabdoviridae_sp
NC_076415|Alpharicinrhavirus_blanchseco
ON812516|Rhabdoviridae_sp
ON812518|Rhabdoviridae_sp
ON812517|Rhabdoviridae_sp
ON812515|Rhabdoviridae_sp
ON812474|Rhabdoviridae_sp
ON812512|Rhabdoviridae_sp
ON812514|Rhabdoviridae_sp
ON812513|Rhabdoviridae_sp
ON812525|Rhabdoviridae_sp
ON812526|Rhabdoviridae_sp
ON812528|Rhabdoviridae_sp
ON812552|Rhabdoviridae_sp
ON812549|Rhabdoviridae_sp
ON746518|Alpharicinrhavirus_huanggang
ON812550|Rhabdoviridae_sp
ON812522|Rhabdoviridae_sp
ON812574|Rhabdoviridae_sp
ON746526|Yanbian_Rhabd_tick_virus_4
ON408168|Alpharicinrhavirus_tahe
ON408167|Alpharicinrhavirus_tahe
ON408169|Alpharicinrhavirus_tahe
OP863293|Manly_virus
ON812554|Rhabdoviridae_sp
ON812480|Rhabdoviridae_sp
ON812557|Rhabdoviridae_sp
ON408164|Alpharicinrhavirus_tahe
ON408165|Alpharicinrhavirus_tahe
ON746537|Yanbian_Rhabd_tick_virus_5
ON746519|Alpharicinrhavirus_nanning
ON812553|Rhabdoviridae_sp
OR868987|Rhabdoviridae_sp
ON812533|Rhabdoviridae_sp
NC_028236|Ledantevirus_kumasi
NC_076186|Rhabdoviridae_sp
MW721925|Alpharicinrhavirus_wuhan
MW721928|Alpharicinrhavirus_wuhan
ON812490|Alpharicinrhavirus_wuhan
ON812493|Alpharicinrhavirus_wuhan
MW721921|Alpharicinrhavirus_wuhan
MW721929|Alpharicinrhavirus_wuhan
MW721924|Alpharicinrhavirus_wuhan
ON812494|Alpharicinrhavirus_wuhan
ON812473|Alpharicinrhavirus_wuhan
MW721930|Alpharicinrhavirus_wuhan
MW721926|Alpharicinrhavirus_wuhan
MW721931|Alpharicinrhavirus_wuhan
MW721927|Alpharicinrhavirus_wuhan
ON812495|Alpharicinrhavirus_wuhan
ON812492|Alpharicinrhavirus_wuhan
ON812491|Alpharicinrhavirus_wuhan
MW721932|Alpharicinrhavirus_wuhan
OR148408|Alpharicinrhavirus_wuhan
OR148426|Alpharicinrhavirus_wuhan
MW721933|Alpharicinrhavirus_wuhan
ON812489|Alpharicinrhavirus_wuhan
ON812487|Alpharicinrhavirus_wuhan
ON812488|Alpharicinrhavirus_wuhan
OM264162|Alpharicinrhavirus_wuhan
OM264163|Alpharicinrhavirus_wuhan
PP471888|Alpharicinrhavirus_wuhan
PP471890|Alpharicinrhavirus_wuhan
PP471892|Alpharicinrhavirus_wuhan
ON812497|Alpharicinrhavirus_wuhan
ON812486|Alpharicinrhavirus_wuhan
OM264160|Alpharicinrhavirus_wuhan
OM264161|Alpharicinrhavirus_wuhan
PP471889|Alpharicinrhavirus_wuhan
PP471891|Alpharicinrhavirus_wuhan
ON812496|Alpharicinrhavirus_wuhan
MG764528|Alpharicinrhavirus_wuhan
MN095536|Alpharicinrhavirus_wuhan
ON812485|Rhipicephalus_associated_rhabdo−like_virus
ON812511|Rhipicephalus_associated_rhabdo−like_virus
ON812580|Rhipicephalus_associated_rhabdo−like_virus
MZ244313|Rhipicephalus_associated_rhabdo−like_virus
ON812510|Rhipicephalus_associated_rhabdo−like_virus
MH814974|Rhipicephalus_associated_rhabdo−like_virus
ON812509|Rhipicephalus_associated_rhabdo−like_virus
ON812508|Rhipicephalus_associated_rhabdo−like_virus
ON812498|Rhipicephalus_associated_rhabdo−like_virus
ON812506|Rhipicephalus_associated_rhabdo−like_virus
ON812503|Rhipicephalus_associated_rhabdo−like_virus
ON812505|Rhipicephalus_associated_rhabdo−like_virus
ON812507|Rhipicephalus_associated_rhabdo−like_virus
ON812504|Rhipicephalus_associated_rhabdo−like_virus
ON812502|Rhipicephalus_associated_rhabdo−like_virus
ON812501|Rhipicephalus_associated_rhabdo−like_virus
ON812499|Rhipicephalus_associated_rhabdo−like_virus
ON746530|Alpharicinrhavirus_qinghai
ON684361|Alpharicinrhavirus_reticulatus
KY385392|Ledantevirus_kanyawara
NC_039020|Ledantevirus_kanyawara
MT325646|Ledantevirus_kanyawara
MT325648|Ledantevirus_kanyawara
MT325650|Ledantevirus_kanyawara
MT325647|Ledantevirus_kanyawara
MT325653|Ledantevirus_kanyawara
MT325652|Ledantevirus_kanyawara
MT325657|Ledantevirus_kanyawara
MT325658|Ledantevirus_kanyawara
MT325659|Ledantevirus_kanyawara
MT325661|Ledantevirus_kanyawara
MT325643|Ledantevirus_kanyawara
MT325644|Ledantevirus_kanyawara
MT325642|Ledantevirus_kanyawara
MT325645|Ledantevirus_kanyawara
MT325654|Ledantevirus_kanyawara
MT325656|Ledantevirus_kanyawara
NC_076494|Ledantevirus_bughendera
NC_076841|Ledantevirus_taiyi
NC_034545|Ledantevirus_elgon
NC_031301|Ledantevirus_wuhan
NC_034548|Ledantevirus_oita
LC717430|Ledantevirus_oita
NC_077171|Ledantevirus_wenzhou
NC_025342|Ledantevirus_kolente
NC_025341|Ledantevirus_fikirini
NC_031305|Ledantevirus_yongjia
ON812534|Ledantevirus_yongjia
ON812478|Ledantevirus_yongjia
ON812537|Ledantevirus_yongjia
ON812535|Ledantevirus_longquan
ON812536|Ledantevirus_longquan
NC_077130|Ledantevirus_longquan
ON812527|Ledantevirus_longquan
NC_034535|Ledantevirus_barur
OM179802|Ledantevirus_barur
NC_034454|Ledantevirus_fukuoka
NC_038276|Ledantevirus_nishimuro
NC_034539|Ledantevirus_nkolbisson
NC_034443|Ledantevirus_ledantec
OQ077988|Ledantevirus_ledantec
OR607590|Odro_virus
NC_034540|Ledantevirus_keuraliba
NC_043538|Ledantevirus_vaprio
OR868176|Bat_ledantevirus
NC_034451|Ledantevirus_kern
NC_076266|Lostrhavirus_hyalomma
OP313011|Lostrhavirus_alxa
NC_075991|Lostrhavirus_lonestar
PP473658|Alpharicinrhavirus_heilongjiang
PP473660|Alpharicinrhavirus_heilongjiang
ON408171|Alpharicinrhavirus_heilongjiang
ON408170|Alpharicinrhavirus_heilongjiang
PP473659|Alpharicinrhavirus_heilongjiang
MF141073|Alpharicinrhavirus_skanevik
OP480464|Alpharicinrhavirus_skanevik
MF141072|Alpharicinrhavirus_skanevik
OP863286|Alpharicinrhavirus_skanevik
NC_031083|Alpharicinrhavirus_huangpi
OK128265|Alpharicinrhavirus_bancrofti
Cluster
Blanchseco
Bole
Guyuan
Huangpi
Hubei
Ledante
Lostrha
Manly
Mononega
Wuhan
